# Commensal bacteria promote type I interferon signaling to maintain immune tolerance in mice

**DOI:** 10.1084/jem.20230063

**Published:** 2023-12-12

**Authors:** Adriana Vasquez Ayala, Chia-Yun Hsu, Renee E. Oles, Kazuhiko Matsuo, Luke R. Loomis, Ekaterina Buzun, Marvic Carrillo Terrazas, Romana R. Gerner, Hsueh-Han Lu, Sohee Kim, Ziyue Zhang, Jong Hwee Park, Paul Rivaud, Matt Thomson, Li-Fan Lu, Booki Min, Hiutung Chu

**Affiliations:** 1 Department of Pathology, University of California, San Diego, La Jolla, CA, USA; 2 Division of Chemotherapy, Kindai University Faculty of Pharmacy, Higashi-osaka, Japan; 3 TUM School of Life Sciences Weihenstephan, ZIEL Institute for Food & Health, Freising-Weihenstephan, Germany; 4 Department of Microbiology and Immunology, Northwestern University Feinberg School of Medicine, Chicago, IL, USA; 5 School of Biological Sciences, University of California, San Diego, La Jolla, CA, USA; 6 Division of Biology, California Institute of Technology, Pasadena, CA, USA; 7 Chiba University-UC San Diego Center for Mucosal Immunology, Allergy and Vaccines, University of California, San Diego, La Jolla, CA, USA; 8 Humans and the Microbiome Program, Canadian Institute for Advanced Research, Toronto, Canada

## Abstract

Type I interferons (IFNs) exert a broad range of biological effects important in coordinating immune responses, which have classically been studied in the context of pathogen clearance. Yet, whether immunomodulatory bacteria operate through IFN pathways to support intestinal immune tolerance remains elusive. Here, we reveal that the commensal bacterium, *Bacteroides fragilis*, utilizes canonical antiviral pathways to modulate intestinal dendritic cells (DCs) and regulatory T cell (Treg) responses. Specifically, IFN signaling is required for commensal-induced tolerance as IFNAR1-deficient DCs display blunted IL-10 and IL-27 production in response to *B. fragilis*. We further establish that IFN-driven IL-27 in DCs is critical in shaping the ensuing Foxp3^+^ Treg via IL-27Rα signaling. Consistent with these findings, single-cell RNA sequencing of gut Tregs demonstrated that colonization with *B. fragilis* promotes a distinct IFN gene signature in Foxp3^+^ Tregs during intestinal inflammation. Altogether, our findings demonstrate a critical role of commensal-mediated immune tolerance via tonic type I IFN signaling.

## Teaser

Commensal bacteria, such as *Bacteroides fragilis*, triggers type I IFN and IL-27 in DCs to influence Tregs through IL27RA signaling. The IFN gene signature in Tregs during inflammation highlights the role of the microbiota in maintaining tonic IFN to promote immune tolerance.

## Introduction

Type I interferons (IFNs) are involved in many essential immune functions, influencing both innate and adaptive immune responses (Trinchieri, 2010; Ivashkiv and Donlin, 2014; McNab et al., 2015). Type I IFNs, namely IFNα and IFNβ, are produced upon sensing microbial products resulting in the expression of interferon-stimulated genes (ISGs). While several hundred ISGs with various known functions have been identified, type I IFN has been primarily studied for its role in antiviral immunity (Schoggins et al., 2011; Schoggins, 2019). This includes recent work that revealed that commensal microbes are involved in maintaining tonic type I IFN necessary to mount an effective antiviral immune response (Abt et al., 2012; Ganal et al., 2012; Yang et al., 2021; Bradley et al., 2019; Wirusanti et al., 2022). Apart from the induction of antiviral ISGs, type I IFN can promote dendritic cell (DC) activation and maturation to enhance antigen presentation to prime adaptive immunity (Honda et al., 2003; Simmons et al., 2012). Further, tonic type I IFN expression is essential for effective T cell responses (Aman et al., 1996; Levings et al., 2001; Bilsborough et al., 2003; Teijaro et al., 2013; Wilson et al., 2013). These studies highlight a potential role for microbial-induced IFN signaling in host immunity beyond antiviral responses. Of particular interest is the divergent effect of type I IFN on immune responses that depend on the context of microbial exposure. Type I IFN responses to microbial pathogens generate a robust antimicrobial and proinflammatory response via activation of distinct ISGs (McNab et al., 2015; Boxx and Cheng, 2016). In contrast, detection of commensal products during homeostatic conditions triggers type I IFN signaling to support anti-inflammatory responses (Lee and Ashkar, 2018). Previous studies also suggest that type I IFN may influence regulatory T cell (Treg) function. Notably, signaling via interferon-α/β receptor 1 (IFNAR1) is required for Treg expansion and suppression of pathogenic T cells during colitis (Lee et al., 2012; Stewart et al., 2013; Kawano et al., 2018). In humans, several genes in the IFN pathway have been associated with inflammatory bowel disease (IBD) susceptibility in genome-wide association studies. *IFNAR1* has been implicated as an IBD risk allele, as have single nucleotide polymorphisms that disrupt the JAK/STAT pathway, resulting in defective IFN production (Jostins et al., 2012). Collectively, these findings support the hypothesis that microbially induced type I IFN signaling plays a role in the maintenance of mucosal homeostasis and immune tolerance.

Clinical and experimental evidence has implicated the gut microbiota in governing host immunity during steady-state and disease (Hooper et al., 2012; Belkaid and Hand, 2014). In particular, the mechanism by which commensal bacteria regulate type I IFN responses to maintain mucosal immunity is of significant interest. Microbiota-induced IFN pathways have been shown to be critical in mounting antiviral resistance in the lung (Steed et al., 2017; Bradley et al., 2019). Moreover, glycolipids from commensal *Bacteroides* have been reported to direct antiviral function via IFNβ expression in DCs (Stefan et al., 2020). In contrast, type I IFN is also involved in the maintenance of Tregs in the gut (Lee et al., 2012; Kole et al., 2013; Nakahashi-Oda et al., 2016). Whether the induction of type I IFN by immunomodulatory commensal bacteria is necessary to maintain intestinal immune tolerance, in addition to driving antiviral responses, is unknown.

Here, we establish that tonic type I IFN is maintained by commensal bacteria and required for tolerogenic immune responses in the gut. Previous work from our group and others established that *Bacteroides fragilis* prime DCs to promote Foxp3^+^ Treg responses to control intestinal inflammation (Shen et al., 2012; Chu et al., 2016). In the present study, we expand on these findings by demonstrating that germ-free (GF) mice are indeed deficient in IFN responses, and colonization with a single commensal bacterium, *B. fragilis*, restores tonic type I IFN in the gut comparable with specific pathogen–free (SPF) mice. We also reveal that select commensal bacteria demonstrate variable induction of IFN signaling in DCs, suggesting this immunomodulatory trait is specialized among certain commensal microbes. Furthermore, our study highlights that *B. fragilis* triggers the production of immunoregulatory cytokines, including IL-10 and IL-27, by DCs through an IFN-dependent manner. This mechanism intricately links *B. fragilis*–induced tonic IFN production in DCs to drive Treg responses via IL-27Rα signaling. Indeed, while investigating *B. fragilis*–mediated gene signatures in Treg cells, we discovered an enrichment of IFN-related genes among intestinal Foxp3^+^ Treg cells, which importantly also include the IL-27 signaling pathway. Our findings demonstrate that commensal bacteria promote intestinal homeostasis through type I IFN signaling.

## Results

### Commensal bacteria direct intestinal type I IFN responses

**Figure 1.**
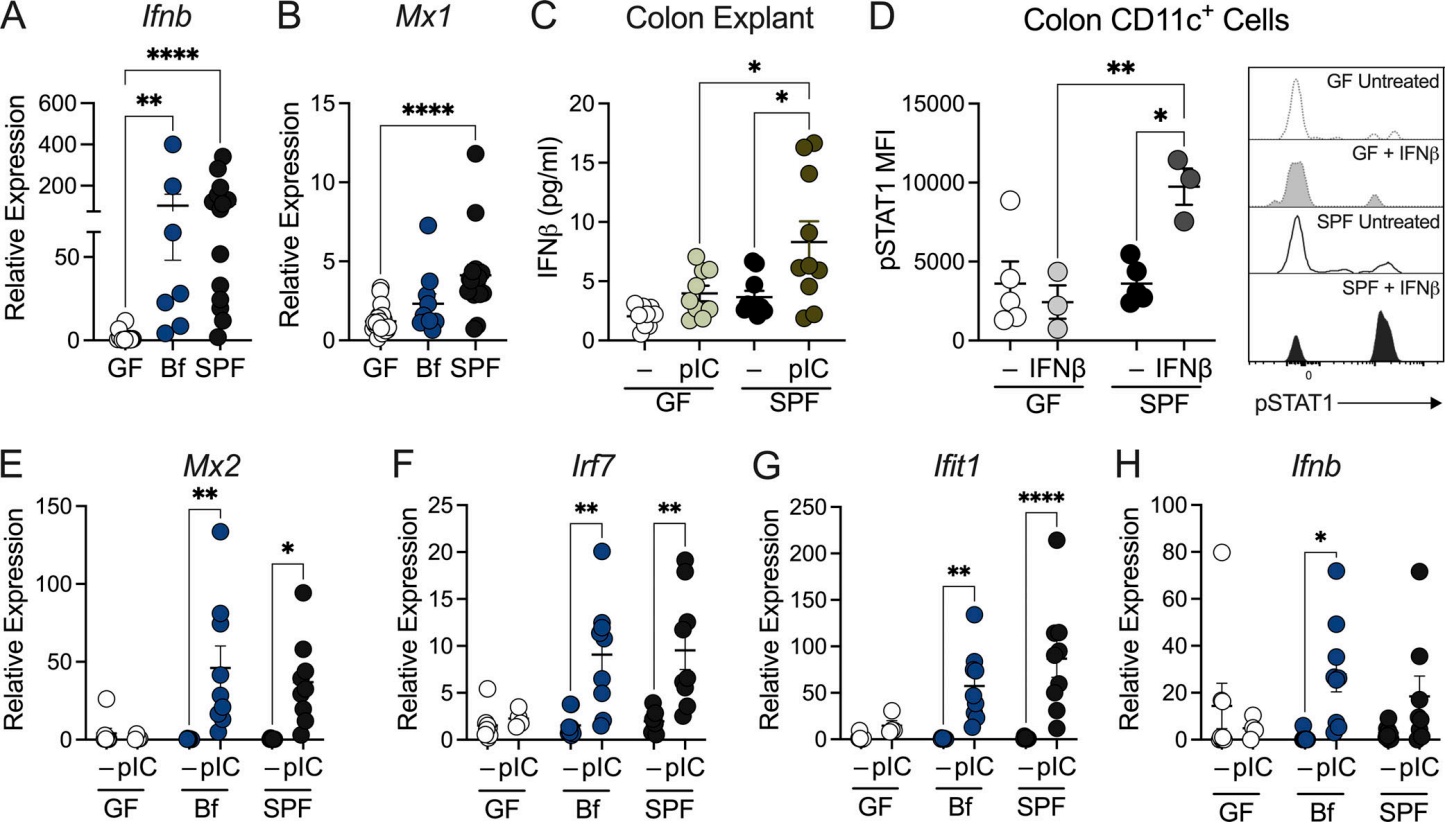
**Commensal bacteria maintain intestinal type I IFN responses. (A and B)** Expression of (A) *Ifnb* and (B) *Mx1* in colon tissue from GF, *B. fragilis*–monocolonized (Bf), and SPF as measured by qRT-PCR relative to β-actin. Each point represents a single mouse. **(C)** GF and SPF colon explants were cultured with or without stimulation of poly I:C (pIC; 2 μg/ml) for 24 h. Supernatant was then collected and measured for IFNβ secretion by ELISA. Each point represents a single mouse. **(D)** cLP cells were isolated from GF and SPF mice and stimulated with IFNβ (25 ng/ml). pSTAT1 was assessed by flow cytometry. Each point represents colons pooled from multiple mice, with *n* = 10 per group. **(E–H)** GF, Bf, and SPF mice were injected (IP) with 100 µg/ml pIC, and colon tissues were harvested 4 h after injection. Gene expression for (E) *Mx2*, (F) *Irf7*, (G) *Ifit1*, and (H) *Ifnb* was measured. Each point represents a single mouse. Data are representative of at least two independent experiments. Statistical significance was determined by Kruskal-Wallis, unpaired *t* test, and two-way ANOVA. P < 0.05 (*), P < 0.01 (**), and P < 0.0001 (****).

**Figure S1.**
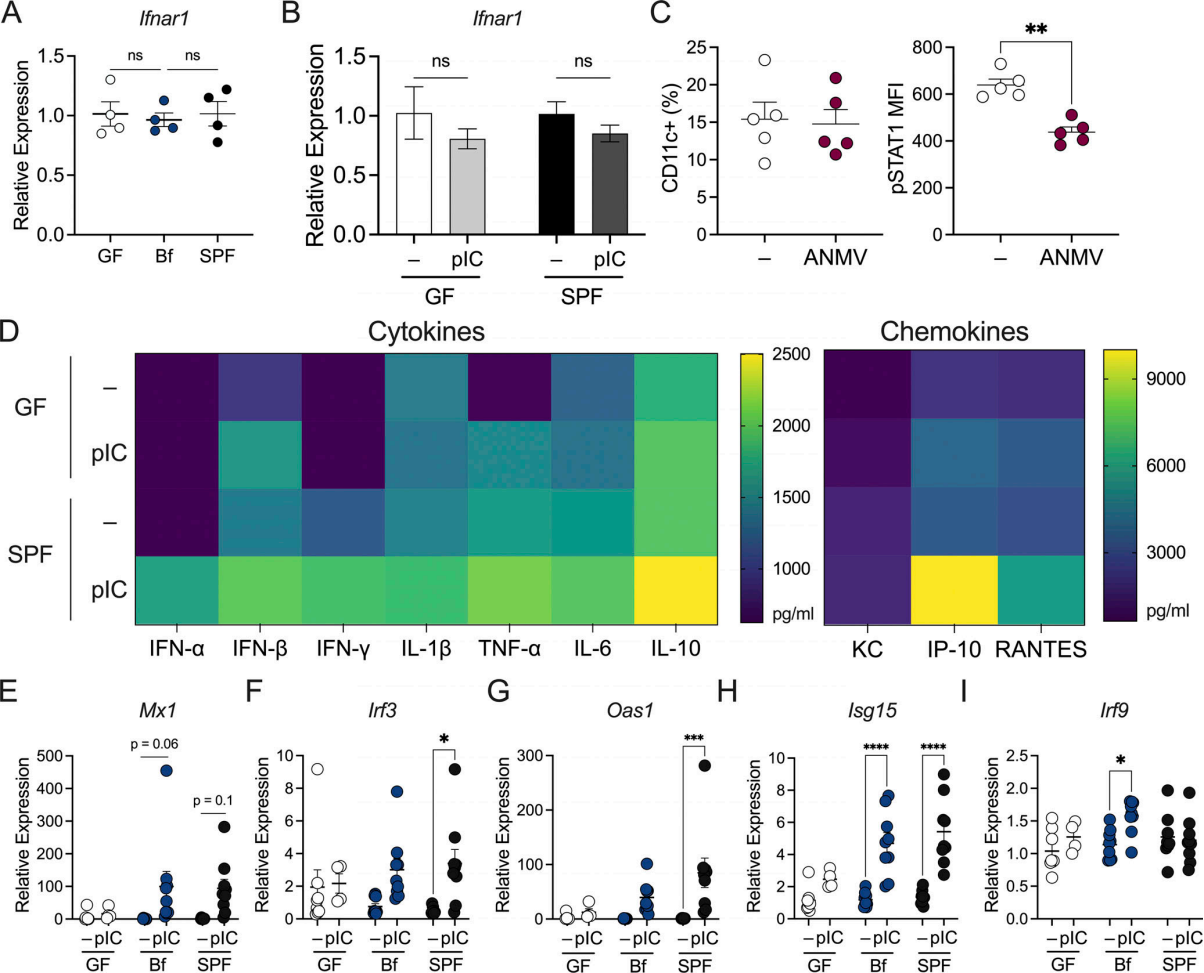
**Gut microbiota are required for the maintenance of type I IFN responses. (A)** Colon tissue from GF, *B. fragilis*–monocolonized (Bf), or SPF mice were harvested and analyzed for expression of *Ifnar1* by qRT-PCR relative to β-actin. **(B)** GF or SPF mice were injected (IP) with 100 µg/mouse poly I:C (pIC) and colon tissues were harvested after 4 h after injection. Gene expression for *Ifnar1* was measured by qRT-PCR relative to β-actin. **(C)** SPF WT C57BL/6 mice were treated with an antibiotic cocktail consisting of ampicillin, metronidazole, neomycin, and vancomycin (ANMV) supplemented with glucose for 2–3 wk, and cLP lymphocytes were isolated and analyzed by flow cytometry to visualize proportions of cLP CD11c^+^ cells and pSTAT1 MFI among CD11c^+^ cells. **(D)** GF and SPF splenocytes were treated with 2 µg/ml pIC for 18 h. Supernatant was collected to measure cytokine and chemokine secretion by multiplex ELISA. **(E–I)** GF, Bf, and SPF mice were injected (IP) with 100 µg/mouse pIC and colon tissues were harvested after 4 h after injection. Gene expression relative to β-actin for (E) *Mx1*, (F) *Irf3*, (G) *Oas1*, (H) *Isg15*, and (I) *Irf9* was measured. Each point represents a single mouse. Data are representative of two experiments. Statistical significance was determined by two-way ANOVA. P < 0.05 (*), P < 0.01 (**), P < 0.001 (***), and P < 0.0001 (****).

Emerging evidence suggests commensal bacteria are important regulators of tonic type I IFN signaling (Sonnenburg et al., 2006; Yamamoto et al., 2012; Schaupp et al., 2020; Domizio et al., 2020; Lam et al., 2021) and are required to mount an effective immune response to pathogens (Abt et al., 2012; Steed et al., 2017; Bradley et al., 2019; Winkler et al., 2020; Erttmann et al., 2022). Given the pleiotropic effects of type I IFN, we examined the association between the microbiota and type I IFN signaling in the gut during steady state by assessing the expression of IFN-related genes in colon tissue of GF and SPF C57BL/6J mice. The presence of a commensal microbial community was required for the induction of *Ifnb* and *Mx1* (an IFN-stimulated gene; ISG) expression in the colon (Fig. 1, A and B). Particularly, monocolonization of GF mice with the commensal bacterium, *B. fragilis*, was sufficient to partially restore IFN-related gene expression in the colon (Fig. 1 A). We next investigated the contribution of the microbiota in priming local intestinal type I IFN responses upon stimulation. Colon explants from GF and SPF mice were treated with polyinosinic–polycytidylic acid (poly I:C), a Toll-like receptor 3 (TLR-3) agonist and potent inducer of IFNβ. While poly I:C induced a significant increase in IFNβ production in SPF colon explants, GF tissues remained unresponsive to poly I:C (Fig. 1 C). We next investigated whether the presence of commensal bacteria influenced IFNAR-dependent signaling in gut DCs. Colonic lamina propria (cLP) cells were isolated from SPF and GF mice and stimulated with recombinant IFNβ ex vivo. Colonic CD11c^+^ cells from SPF mice responded to IFNβ stimulation via increased phosphorylation of signal transducer and activator of transcription 1 (STAT1), as measured by flow cytometry (Fig. 1 D). Conversely, pSTAT1 expression in colonic CD11c^+^ cells from GF mice remained unchanged in colonic CD11c^+^ cells from GF mice following IFNβ stimulation, suggesting impaired IFNAR signaling in mice lacking commensal bacteria despite equivalent expression of *Ifnar1* (Fig. 1 D and Fig. S1, A and B). Antibiotic cocktail treatment of SPF mice also led to diminished pSTAT1 expression (Fig. S1 C). These CD11c^+^ cells encompass both DCs and macrophages, and we speculate both cell types play a role in sensing commensal bacteria in the gut. Together, these findings establish that commensal bacteria direct IFN signaling and upregulation of pSTAT1 in gut CD11c^+^ immune cells. To investigate whether this IFN defect in the intestinal environment extends to systemic compartments, splenocytes from GF and SPF mice were treated ex vivo with poly I:C. As expected, at baseline, SPF splenocytes expressed higher levels of type I IFN, as well as other cytokines and chemokines, compared with GF (Fig. S1 D). In contrast, GF splenocytes failed to mount a robust response to poly I:C stimulation compared with SPF. Moreover, no induction of IFNα and IFNɣ (a type II IFN) was observed upon poly I:C treatment of GF splenocytes, while poly I:C induced IFNβ in GF splenocytes, secretion was limited and equivalent to untreated SPF cells (Fig. S1 D). To confirm the requirement of commensal bacteria for type I IFN response in the intestinal environment in vivo, GF, *B. fragilis*–monocolonized, and SPF mice were treated with poly I:C by intraperitoneal (IP) injection and evaluated for IFN responsiveness. SPF mice treated with poly I:C demonstrated significant expression of type I IFN–related genes (Fig. 1, E–G; and Fig. S1, F–H). In contrast, GF mice injected with poly I:C showed no response in comparison to the PBS control (Fig. 1, E–H; and Fig. S1, E–I), consistent with ex vivo studies (Fig. 1, A–D). To verify whether this IFN defect in GF mice can be restored with commensal bacteria, we colonized GF mice with *B. fragilis*. Indeed, poly I:C treatment of *B. fragilis* mice led to a significant induction of IFNβ and IFN-related genes, indicating that the presence of commensal bacterium is sufficient to restore homeostatic type I IFN responses (Fig. 1, E–H; and Fig. S1, H and I). These data establish that commensal bacteria are critical in restoring and maintaining type I IFN responses in intestinal tissues.

### Select commensal bacteria induce IFN signaling to promote tolerogenic responses in DCs

**Figure 2.**
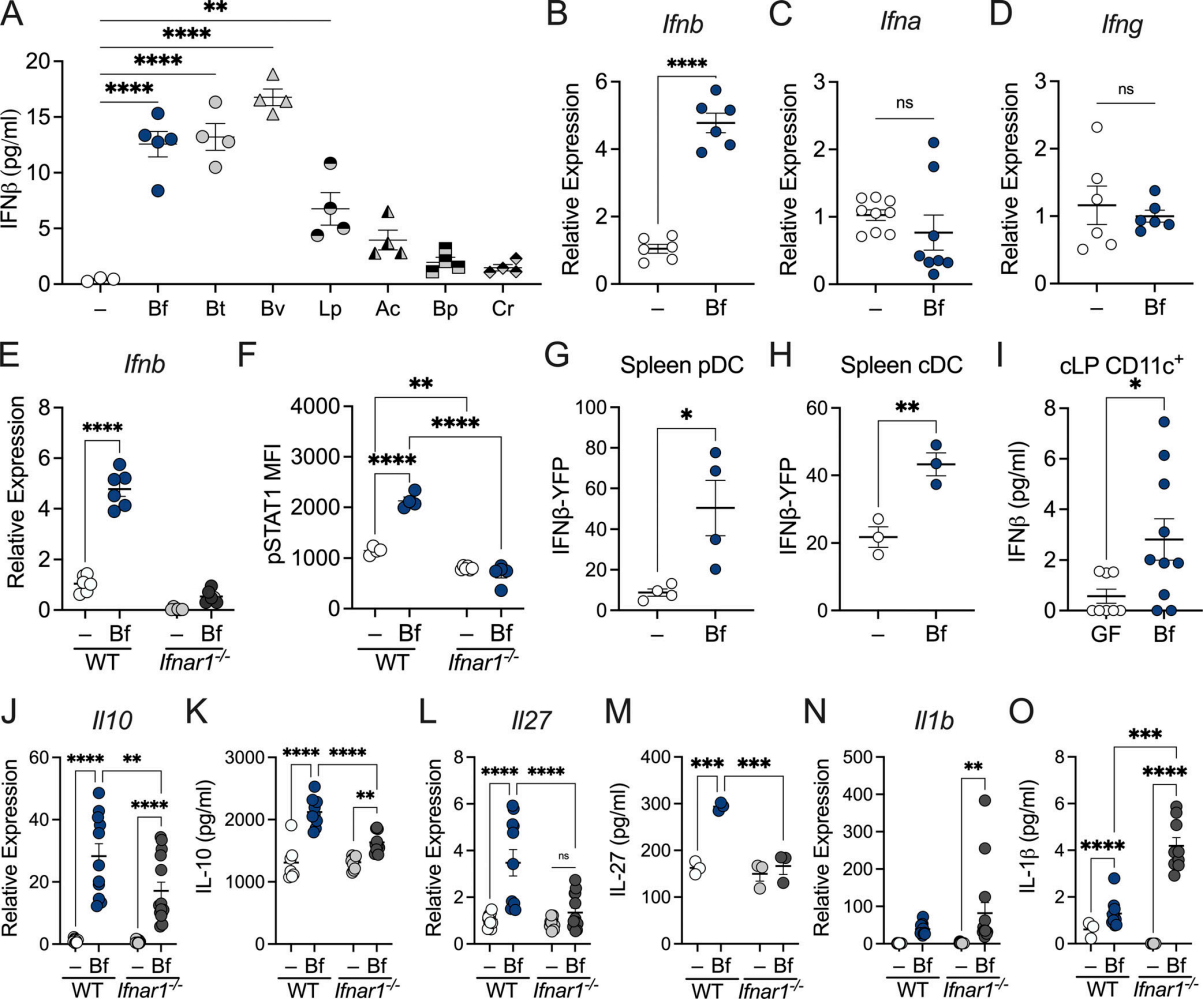
**Select commensal bacteria induce IFN signaling to promote tolerogenic responses in DCs. (A)** BMDCs from SPF mice were treated with *B. fragilis* (Bf), *B. thetaiotaomicron* (Bt), *B. vulgatus* (Bv), *L. plantarum* (Lp), *A. caccae* (Ac), *B. producta* (Bp), and *C. ramosum* (Cr) for 18 h. Supernatant was collected and IFNβ production was measured by ELISA. **(B–D)** BMDCs were treated with Bf for 18 h and expression of (B) *Ifnb*, (C) *Ifna*, and (D) *Ifng* were measured relative to β-actin by qRT-PCR. **(E and F)** WT and *Ifnar1*^−/−^ BMDCs were pulsed with Bf for 18 h. Cells were harvested and analyzed by qRT-PCR for gene expression of (E) IFNβ and stained for (F) flow cytometry analysis of pSTAT1 in CD11c^+^ DCs. **(G and H)** Splenocytes from IFNβ-YFP reporter mice were treated with Bf ex vivo for 18 h and mean fluorescent intensity (MFI) of IFNβ-YFP was assessed in (G) plasmacytoid dendritic cells (pDCs) and (H) conventional dendritic cells (cDCs) by flow cytometry. **(I)** cLP cells were isolated and enriched for CD11c^+^ cells from GF and Bf-monocolonized mice and cultured for 18 h. Supernatant was collected and IFNβ production was measured by ELISA. Each point represents colons pooled from five mice per point and represents *n* = 30 for each group. **(J–O)** WT and IFNAR-deficient BMDCs were pulsed with Bf for 18 h. Cells were harvested and analyzed by qRT-PCR for gene expression of (J) *Il10*, (L) *Il27p28*, and (N) *Il1b* relative to β-actin. Supernatant from BMDC cultures was collected and cytokine secretion was measured for (K) IL-10, (M) IL-27p28, and (O) IL-1β by ELISA. Data are representative of at least two independent experiments. Statistical analysis was determined by unpaired *t* test and two-way ANOVA. P < 0.05 (*), P < 0.01 (**), P < 0.001 (***), and P < 0.0001 (****).

**Figure S2.**
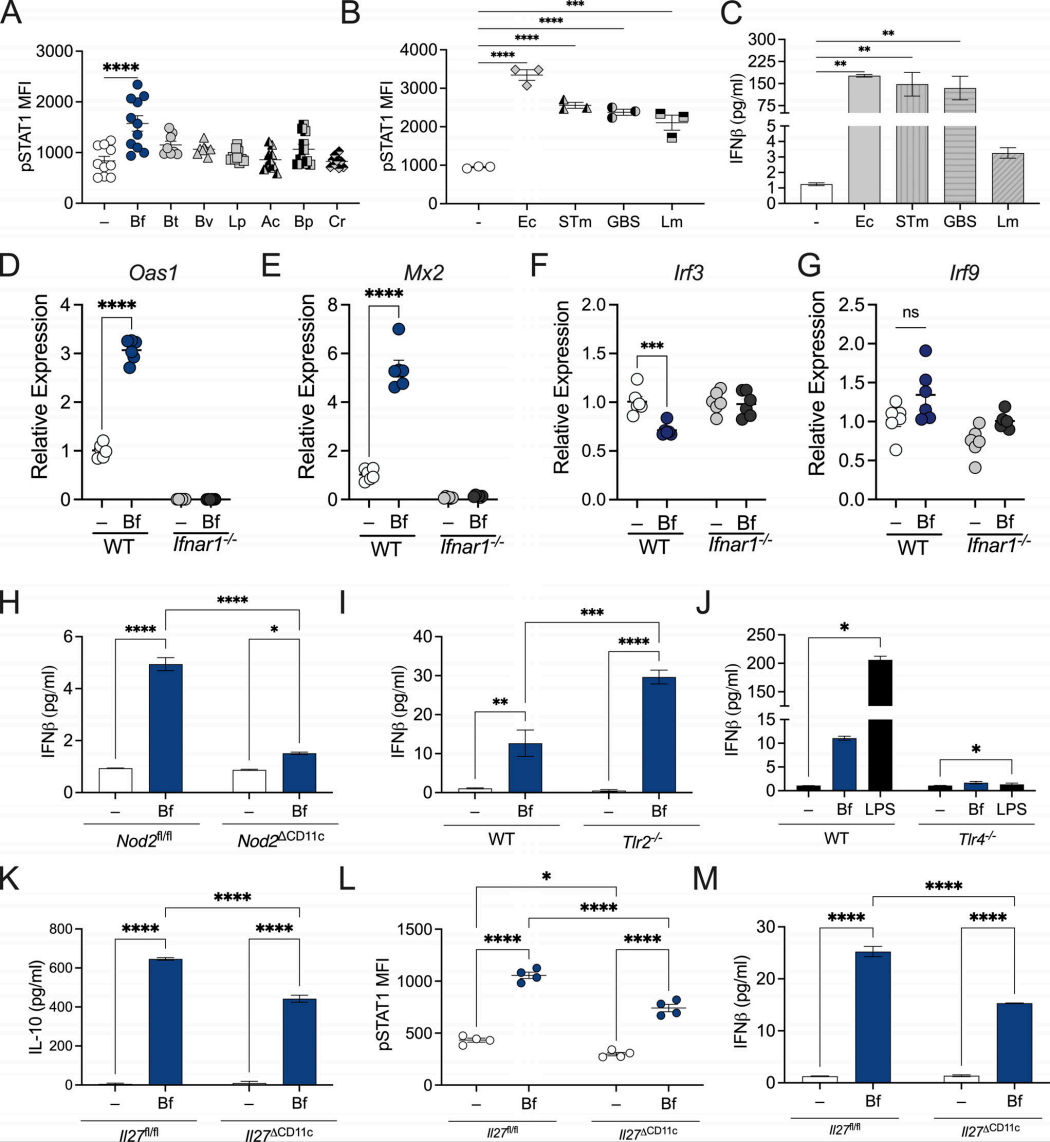
**DC-signaling pathways mediate tonic type I IFN and tolerogenic cytokine responses. (A)** BMDCs from WT SPF mice were treated with *B. fragilis* (Bf), *B. thetaiotaomicron* (Bt), *B. vulgatus* (Bv), *L. plantarum* (Lp), *A. caccae* (Ac), *B. producta* (Bp), and *C. ramosum* (Cr) for 18 h. Cells were stained and pSTAT1 among CD11c^+^ DCs were analyzed by flow cytometry. **(B–C)** BMDCs from WT SPF mice were treated with *E. coli* (Ec), *S. enterica* serovar Typhimurium (STm), Group B *Streptococcus* (GBS), and *L. monocytogenes* (Lm) for 18 h and (B) cells were stained and pSTAT1 among CD11c^+^ DCs was analyzed by flow cytometry and (C) supernatant was collected and measured for IFNβ by ELISA. **(D–G)** WT and IFNAR1-deficient BMDCs were pulsed with Bf for 18 h. Cells were harvested and analyzed by qRT-PCR for expression of (D) *Oas1*, (E) *Mx2*, (F) *Irf3*, and (G) *Irf9*. **(H)** BMDCs from *Nod2*^fl/fl^ and *Nod2*^∆CD11c^ mice were treated with Bf or 100 ng/ml of muramyl dipeptide for 18 h, and supernatants from BMDC cultures were collected and IFNβ secretion was measured by ELISA. **(I)** BMDCs from WT and *Tlr2*^−/−^ mice were treated with Bf or 100 ng/ml of Pam3CSK4 (PAM) for 18 h, and supernatants from BMDC cultures were collected and IFNβ secretion was measured by ELISA. **(J)** BMDCs from WT and *Tlr4*^−/−^ mice were treated with Bf or 100 ng/ml of LPS for 18 h, and supernatants from BMDC cultures were collected and IFNβ secretion was measured by ELISA. **(K–M)** BMDCs from *Il27*^fl/fl^ and *Il27*^∆CD11c^ mice were treated with Bf for 18 h. **(K)** Supernatants from BMDC cultures were collected and IL-10 secretion was measured by ELISA. **(L)** Cells were stained for pSTAT1 and analyzed by flow cytometry. **(M)** Supernatants from BMDC cultures were collected and IFNβ secretion was measured by ELISA. Data are representative of two experiments. Statistical analysis was determined by unpaired *t* test and two-way ANOVA. P < 0.05 (*), P < 0.01 (**), P < 0.001 (***), and P < 0.0001 (****).

DCs establish and maintain the local gut immune milieu by sampling luminal contents, including bacteria (Rescigno and Sabatino, 2009). To gain insight into the impact of commensal-derived type I IFN on DC responses, we first sought to assess the levels of IFNβ induction by commensal bacteria. We demonstrate that DCs treated with *B. fragilis* and other *Bacteroides* species, *Bacteroides thetaiotaomicron* and *Bacteroides vulgatus*, drive IFNβ secretion and pSTAT1 induction, while *Lactobacillus plantarum* induced modest IFNβ production (Fig. 2 A and Fig. S2 A). In contrast, other commensal bacteria tested did not support significant IFNβ induction, suggesting some degree of specificity in their activity (Fig. 2 A and Fig. S2 A). Pathogenic bacteria, on the other hand, induced pSTAT1 expression and IFNβ production (Fig. S2, B and C) at much greater levels than *B. fragilis* and other commensals tested. These variations in type I IFN induction by both commensal and pathogenic bacteria may underlie the diverse immunomodulatory effects observed downstream of recognition.

Since monocolonization with *B. fragilis* effectively restored type I IFN responses in vivo (Fig. 1) and yielded robust type I IFN induction among DCs (Fig. 2 A), we employed *B. fragilis* as a model commensal to delve deeper into the role of IFN responses in mediating immune tolerance. Treatment of bone marrow–derived DCs (BMDCs) with *B. fragilis* induced expression of IFNβ (Fig. 2 B), but not IFNα or IFNɣ (Fig. 2, C and D), consistent with in vivo findings (Fig. 1). As expected, this induction was lost in *Ifnar1*^−/−^ DCs, along with a decrease in pSTAT1 expression (Fig. 2, E and F) in comparison with wild-type (WT) DCs. Additionally, *B. fragilis* significantly induced type I IFN–related genes in WT DCs (e.g., *Ifnb*, *Oas1*, and *Mx2*), whereas *Ifnar1*^−/−^ DCs remained unresponsive to *B. fragilis* (Fig. 2 E and Fig. S2, D and E). Further, *B. fragilis* does not influence expression of other IFN-responsive genes (e.g., *Irf3*, *Irf9*) by WT DCs (Fig. S2, F and G). Next, we leveraged the *IFNb*^mob^EYFP reporter mouse to examine *B. fragilis*–induced IFNβ among conventional and plasmacytoid DCs. Treatment with *B. fragilis* elevated IFNβ-YFP expression in both DC subsets (Fig. 2, G and H). We then examined whether colonization with *B. fragilis* induced IFN expression in colonic DCs. To assess the extent of IFNβ produced by intestinal DCs primed by *B. fragilis* in vivo, we isolated colonic CD11c^+^ cells from GF and *B. fragilis*–monocolonized mice and measured baseline IFNβ secretion. Indeed, colonic lamina propria CD11c^+^ cells from *B. fragilis*–monocolonized mice produced higher levels of IFNβ compared with GF mice (Fig. 2 I).

**Figure S3.**
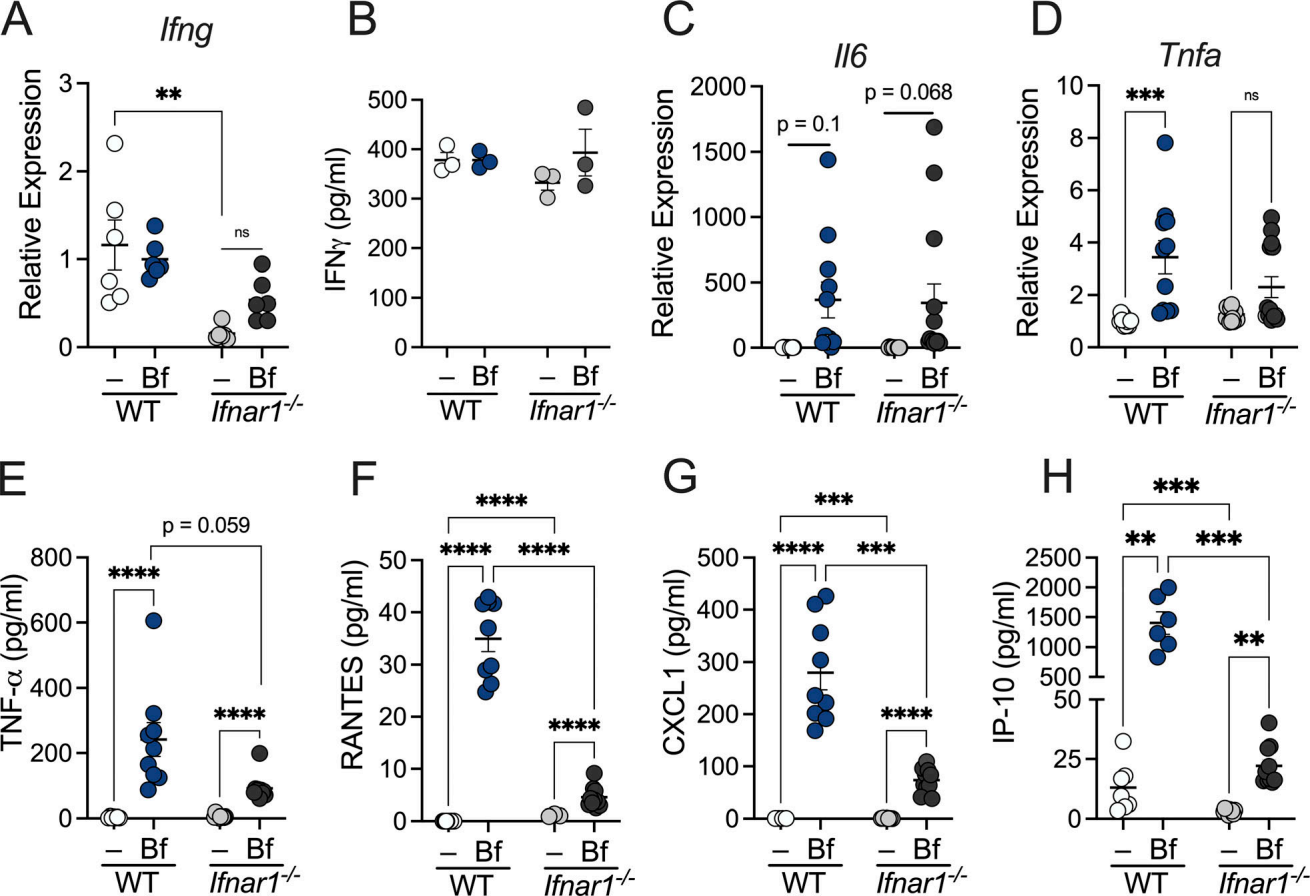
**IFNAR signaling in DCs is required for *B. fragilis*–induced immune responses.** WT and IFNAR1-deficient BMDCs were pulsed with *B. fragilis* for 18 h. **(A, C, and D)** Cells were harvested and analyzed by qRT-PCR for expression of (A) *Ifng*, (C) *Il6*, and (D) *Tnfa* relative to β-actin. **(B and E–H)** Supernatant from BMDC cultures were collected and protein secretion was measured for (B) IFNγ, (E) TNF-α, (F) RANTES, (G) CXCL1, and (H) IP-10 by ELISA. Data are representative of two experiments. Statistical significance was determined by two-way ANOVA. P < 0.01 (**), P < 0.001 (***), and P < 0.0001 (****).

It has been well documented that *B. fragilis* primes DCs to foster immune tolerance (Shen et al., 2012; Chu et al., 2016). However, the precise signaling mechanisms underlying this immune regulation remain incompletely understood. Our observations thus far have revealed a distinct pattern of induction among type I IFN activity in *B. fragilis*–primed DCs. To investigate the key receptors that initiate this pathway, we sought to determine which pattern recognition receptors *B. fragilis* engages to drive type I IFN responses in BMDCs. Previous studies with *B. fragilis* demonstrated that defects in TLR2 (Round et al., 2011; Shen et al., 2012) and NOD2 (Chu et al., 2016) signaling led to impaired Treg responses. We observed *B. fragilis*–induced IFNβ production required signaling via NOD2, but not TLR2 (Fig. S2, H and I). Additionally, we examined the role of TLR4 and revealed reduced IFN responses to *B. fragilis* in TLR4-deficient BMDCs (Fig. S2 J). These findings are consistent with published reports demonstrating TLR4 agonists, but not TLR2, stimulate IFNβ-induced pSTAT1 expression (Fig. S2, I and J; Toshchakov et al., 2002; Stefan et al., 2020). We also previously reported a shift in the cytokine milieu in DCs defective in microbial sensing (Chu et al., 2016), demonstrating a significant decrease in expression of IL-10, which is required for induction of tolerogenic Tregs (Shen et al., 2012). Thus, we investigated how type I IFN deficiency may alter downstream cytokine production. We observed a significant reduction in IL-10 expression and production among *Ifnar1*^−/−^ BMDCs compared with *B. fragilis*–treated WT BMDCs (Fig. 2, J and K). The induction of IL-10 by *B. fragilis* has been well established (Round et al., 2011; Shen et al., 2012; Chu et al., 2016); however, this data reveals the essential role of commensal-induced IFN signaling among DCs to promote tolerogenic responses. We further demonstrate here that *B. fragilis* also stimulates the production of IL-27 in DCs, and this response is abrogated upon IFNAR1 deletion (Fig. 2, L and M). Moreover, we observed a significant reduction in IL-10 production in *Il27*^−/−^ DCs treated with *B. fragilis*, suggesting IFN-induced IL-27 is necessary for commensal-mediated immune homeostasis (Fig. S2 K). Indeed, IL-27 is documented as an immunoregulatory cytokine that targets Tregs to mediate anti-inflammatory activity (Kim et al., 2019; Nguyen et al., 2019), while suppressing IL-17–producing CD4^+^ T cells (Batten et al., 2006). Given that the signaling of IL-27 and type I IFN are intricately linked (Stumhofer et al., 2007; Amsden et al., 2022), we investigated the requirement of IL-27 in *B. fragilis*–mediated type I IFN activity among DCs. We observed *B. fragilis*–induced expression of pSTAT1 (Fig. S2 L) and secretion of IFNβ (Fig. S2 M) relied upon IL-27 production in DCs. These data suggest induction of IFN by *B. fragilis* drives IL-27 expression, which further reciprocates type I IFN signaling. Considering the reduction in IL-10 and IL-27 upon type I IFN deficiency, we investigated how proinflammatory responses may be affected. Notably, the decreased anti-inflammatory response observed is paired with an increase in IL-1β upon *B. fragilis* treatment of *Ifnar1*^−/−^ BMDCs (Fig. 2, N and O). *B. fragilis* treatment of BMDCs did not alter levels of proinflammatory IFNɣ, indicating selectivity in cytokine regulation by commensal microbes (Fig. 2 D; and Fig. S3, A and B). Consistent with these observations, we report extensive dysregulation of cytokine and chemokine production in *B. fragilis*–treated *Ifnar1*^−/−^ BMDCs (Fig. S3, C–H). Collectively, these data establish a critical role for IFN signaling in DCs to induce immunoregulatory responses upon sensing commensal bacteria.

### *B. fragilis* induces type I IFN expression in DCs to coordinate Treg responses

**Figure 3.**
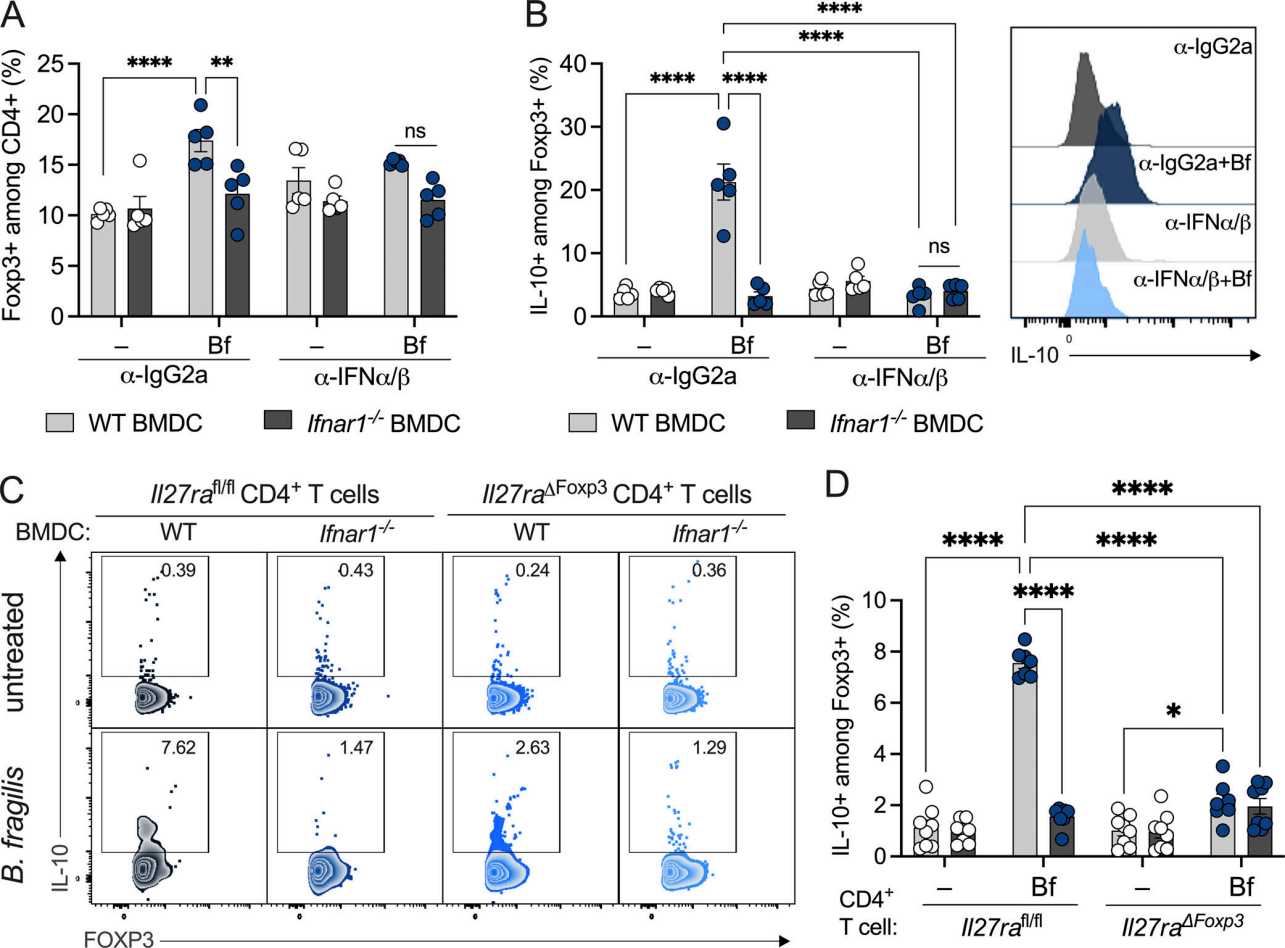
**Type I IFN signaling in DCs is required to promote Treg responses.** WT and *Ifnar1*^−/−^ BMDCs were untreated (−) or treated with *B. fragilis* (Bf) cocultured with WT CD4^+^ T cells. **(A and B)** Cells were treated with 10 µg/ml of IFNα/β neutralizing antibodies or isotype control (IgG2a) every 24 h and then stained and analyzed by flow cytometry for (A) Foxp3^+^ Tregs and (B) IL-10–producing Foxp3^+^ Tregs. **(C and D)** WT and *Ifnar1*^−/−^ BMDCs were untreated (−) or treated with Bf cocultured with *Il27ra*^fl/fl^ or *Il27ra*^∆Foxp3^ CD4^+^ T cells. Cells were stained and analyzed by flow cytometry for IL-10–producing Foxp3^+^ Tregs. (C) Representative plots are shown and (D) proportions of IL-10–producing Foxp3^+^ Tregs are quantified. Data are representative of at least two independent experiments. Statistical significance was determined by two-way ANOVA. P < 0.05 (*), P < 0.01 (**), and P < 0.0001 (****).

We hypothesize that this skewed proinflammatory environment driven by IFNAR-deficient DCs would restrain downstream Treg responses. To test this hypothesis, WT and *Ifnar1*^−/−^ BMDCs were treated with *B. fragilis* and cocultured with WT CD4^+^ T cells. Indeed, *B. fragilis*–pulsed WT DCs supported the induction of CD4^+^ Foxp3^+^ Tregs (Fig. 3 A) and production of IL-10 (Fig. 3 B). However, *B. fragilis*–treated *Ifnar1*^−/−^ BMDCs resulted in a significant reduction of IL-10^+^ Treg populations, indicating IFN signaling in DCs is necessary for the induction of anti-inflammatory T cell. We next assessed whether neutralization of type I IFN phenocopies the Treg defect observed in *B. fragilis*–treated *Ifnar1*^−/−^ DCs. The induction of Treg and IL-10 production by *B. fragilis* was abrogated upon addition of neutralizing antibodies for IFNα and IFNβ in DC:T cell cocultures compared with isotype controls (Fig. 3, A and B). This suggests IFNα and/or IFNβ function are critical signals in mediating *B. fragilis*–induced Tregs.

**Figure S4.**
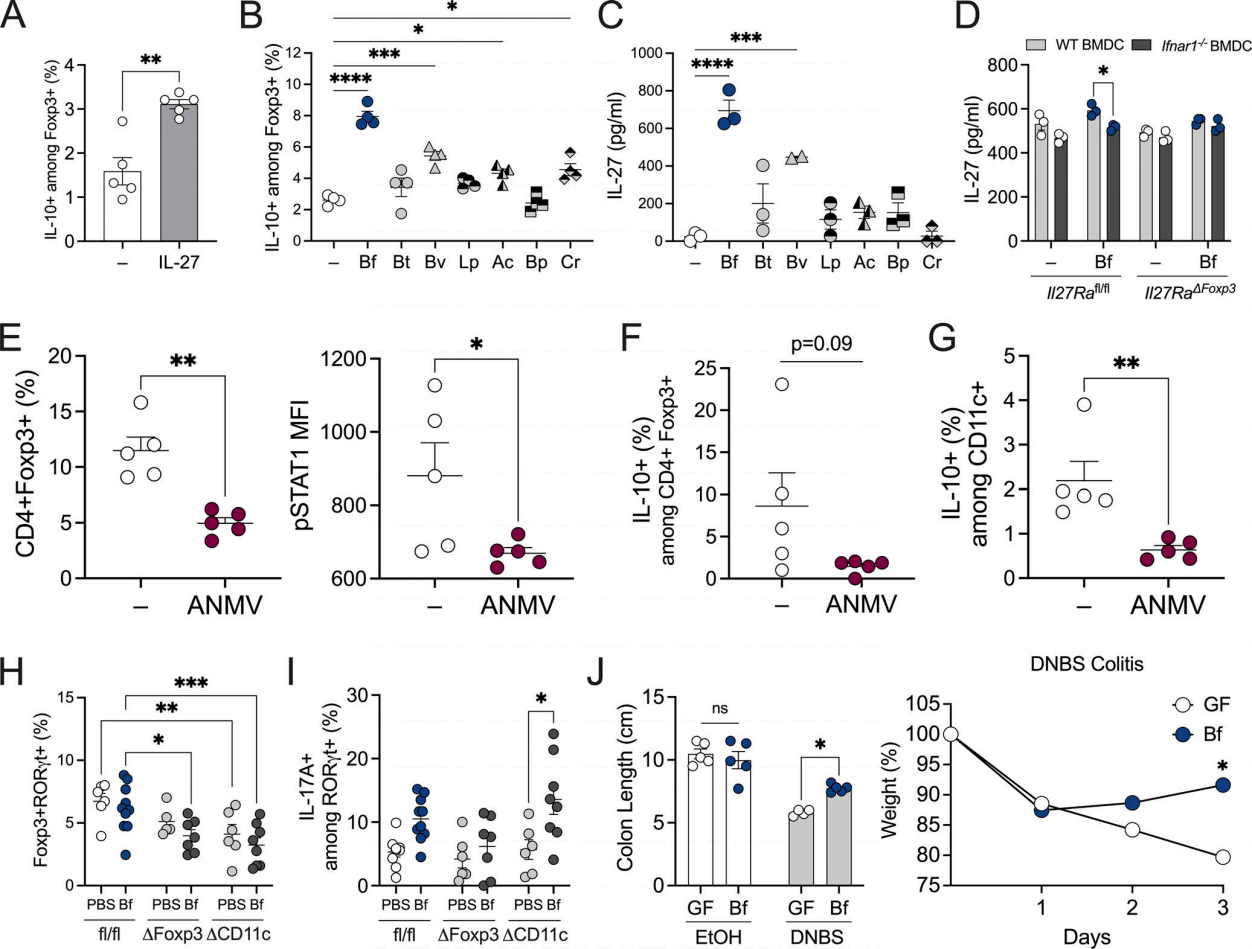
**Type I IFN signaling facilitates commensal-induced Treg responses. (A)** WT BMDCs were untreated (−) or treated with IL-27 (50 ng/ml) cocultured with WT T cells and then stained and analyzed by flow cytometry for IL-10 production among Foxp3^+^ Tregs. **(B)** WT BMDCs were cocultured with WT T-cells with or without *B. fragilis* (Bf), *B. thetaiotaomicron* (Bt), *B. vulgatus* (Bv), *L. plantarum* (Lp), *A. caccae* (Ac), *B. producta* (Bp), and *C. ramosum* (Cr) treatment for 72 h. Cells were then stained to visualize IL-10^+^ Foxp3^+^ Tregs by flow cytometry. **(C)** WT BMDCs were pulsed with Bf, Bt, Bv, Lp, Ac, Bp, and Cr for 18 h. Supernatant was collected and measured for IL-27 production by ELISA. **(D)** WT and IFNAR1-deficient BMDCs were untreated (−) or treated with Bf cocultured with *Il27ra*^fl/fl^ or *Il27ra*^∆Foxp3^ CD4^+^ T cells. Supernatant was collected and measured for IL-27 by ELISA. **(E–G)** SPF WT C57BL/6 mice were treated with an antibiotic cocktail consisting of ampicillin, metronidazole, neomycin, and vancomycin (ANMV) supplemented with glucose for 2–3 wk, and cLP lymphocytes were isolated and analyzed by flow cytometry to visualize proportions of (E) Foxp3^+^ among CD4^+^ T cells and pSTAT1 MFI among Foxp3^+^ CD4^+^ T-cells, (F) IL-10^+^ among Foxp3^+^ CD4^+^ T cells, and (G) IL-10^+^ among cLP CD11c^+^ cells. Each point represents a single mouse. **(H and I)** *Ifnar1*^*fl/f*^, *Ifnar1*^∆CD11c^, and *Ifnar1*^∆Foxp3^ mice were gavaged with either PBS or Bf for 2 wk and proportions of (H) CD4^+^ Foxp3^+^ RORgt^+^ Tregs and (I) RORgt^+^ IL-17A^+^ cells were assessed by flow cytometry. **(J)** 4–6-wk-old female GF Foxp3^hCD2^/IL-10^Venus^ mice were orally gavaged with a single dose of Bf (10^8^ CFU resuspended in sterile PBS). 2 wk later mice were subjected to 5% DNBS colitis or EtOH control for 3 d, and colon length and change in weight percentage were recorded. Data are representative of at least two independent experiments. Statistical analysis was determined by unpaired *t* test and two-way ANOVA. P < 0.05 (*), P < 0.01 (**), P < 0.001 (***), and P < 0.0001 (****).

Previous studies have demonstrated a critical role for IL-27 in supporting IL-10 expression among Tregs (Awasthi et al., 2007; Pot et al., 2009; Kim et al., 2019; Nguyen et al., 2019). In particular, IL-27 has been shown to initiate a transcriptional network that facilitates IL-10 expression among CD4^+^ T cell subsets (Zhang et al., 2020). Further corroborating these studies, we observed marked upregulation of IL-10 expression among Foxp3^+^ Tregs upon IL-27 stimulation (Fig. S4 A). Notably, treatment with commensal bacteria yielded a similar trend in the production of IL-27 and IL-10 among BMDCs and Tregs, respectively, suggesting that commensal-induced IL-27 may play a role in directing Treg responses (Fig. S4, B and C). Given the IFN-dependent induction of IL-27 by *B. fragilis* (Fig. 2, L and M), we hypothesized that IL-27 signals directly on Foxp3+ Tregs to promote tolerogenic function. The IL-27 receptor consists of two subunits, IL-27Rα and glycoprotein 130 (gp130). To elucidate the significance of IL-27 signaling in Foxp3^+^ Tregs, we employed *Il27ra*^fl/fl^ × Foxp3-Cre (*Il27ra*^∆Foxp3^) mice (Do et al., 2017). As expected, coculture of *Il27ra*^fl/fl^ CD4^+^ T cells with *B. fragilis*–pulsed WT DCs led to the induction of IL-10 among CD4^+^ Foxp3^+^ Tregs. However, this induction was markedly diminished when cocultured with *Il27ra*^∆Foxp3^ CD4^+^ T cells (Fig. 3, C and D). Furthermore, when we performed these studies with *Ifnar1*^−/−^ DCs, we again observed a significant reduction in IL-10–producing Foxp3+ Tregs in response to *B. fragilis*, with both *Il27ra*^fl/fl^ and *Il27ra*^∆Foxp3^ CD4^+^ T cells (Fig. 3, C and D). Comparable levels of IL-27 were detected across the various DC:T cell coculture conditions, although a reduction in IL-27 was observed in CD4^+^ T cells cocultured with *B. fragilis*–pulsed *Ifnar1*^−/−^ DCs (Fig. S4 D). This finding aligns with our observation that the induction of IL-27 by *B. fragilis* was lost in *Ifnar1*^−/−^ BMDCs (Fig. 2, L and M). Altogether, these data suggest a model wherein commensal bacteria promote the production of IL-27 from DCs through an IFN-dependent pathway, which in turn signals via IL-27Rα in Foxp3^+^ Tregs to orchestrate immune tolerance.

### Commensal colonization drives IFN gene signature in intestinal Tregs

**Figure 4.**
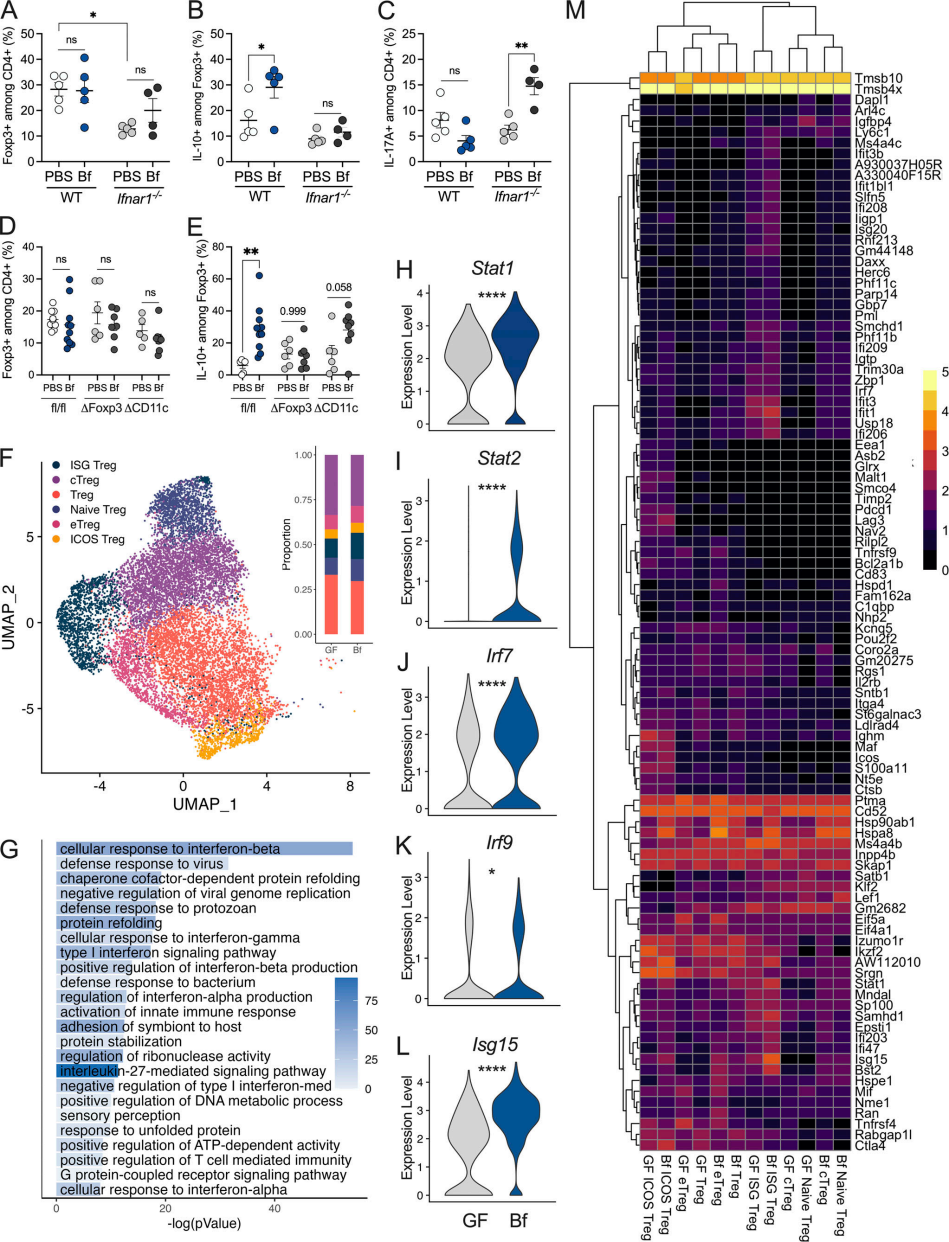
**Bacterial colonization induces type I IFN signature in intestinal Tregs. (A–C)** WT and *Ifnar1*^*−/−*^ SPF mice were orally gavaged with either sterile PBS or live *B. fragilis* (Bf; 2 × 10^8^ CFUs) for 2 wk and proportions of (A) CD4^+^ Foxp3^+^ Tregs, (B) CD4^+^ Foxp3^+^ IL10^+^ Tregs, and (C) CD4^+^ Foxp3^−^ IL17A^+^ T cells in cLP were assessed by flow cytometry. **(D and E)** *Ifnar1*^*fl/fl*^, *Ifnar1*^∆Foxp3^, and *Ifnar1*^∆CD11c^ SPF mice were orally gavaged with either sterile PBS or live Bf (2 × 10^8^ CFUs) for 2 wk and proportions of (D) CD4^+^ Foxp3^+^ Tregs and (E) Foxp3^+^ IL-10^+^ Tregs were assessed by flow cytometry. **(F–M)** 4–6-wk-old female GF Foxp3^hCD2^/IL-10^Venus^ mice were orally gavaged with a single dose of Bf (10^8^ CFUs). 2 wk later, mice were subjected to 5% DNBS colitis for 3 d and MLNs were harvested, and single suspensions were prepared. CD4^+^hCD2^+^ cells were enriched and subjected to single-cell RNA sequence analysis. **(F)** UMAP projection of Tregs from MLNs from GF and Bf mice showing six clusters of Treg subpopulations: ISG Treg, central Treg (cTreg), Treg, naive Treg, effector Treg (eTreg), and ICOS Treg. Each dot corresponds to a single cell, colored according to cell type. Inset, bar plot of the proportions of each Treg subpopulation in GF and Bf MLNs. **(G)** GO enrichment analysis of ISG Tregs in Bf mice relative to GF controls. Gene expression of (H) *Stat1*, (I) *Stat2*, (J) *Irf7*, (K) *Irf9*, and (L) *Isg15* in ISG Tregs of GF and Bf mice. **(M)** Heatmap showing the top 100 differentially expressed genes in the six Treg subpopulations in GF and Bf MLNs. The dendrogram is based on a hierarchical cluster analysis of Euclidean distances. Each point represents a single mouse. Statistical significance was determined by two-way ANOVA. P < 0.05 (*), P < 0.01 (**), and P < 0.0001 (****).

Given the requirement of type I IFN signaling for *B. fragilis*–induced Treg responses in vitro, we investigated the consequence of IFNAR1 deficiency in SPF mice in vivo. Proportions of Foxp3^+^ Tregs in the cLP were reduced in *Ifnar1*^−/−^ mice (Fig. 4 A), consistent with previous work that reported loss of Treg induction in *Rag1*^*−/−*^ recipients upon transfer of *Ifnar1*^*−/−*^ Tregs (Lee et al., 2012). As expected, *B. fragilis* treatment of WT SPF mice induced IL-10–producing Foxp3^+^ Tregs compared with PBS-treated controls (Fig. 4 B). In contrast, *B. fragilis*–induced Treg responses were abolished in *Ifnar1*^−/−^ SPF mice (Fig. 4 B). This dysregulation of Treg function is often associated with an increased frequency of pathogenic T cell subsets, such as Th1 or Th17. Given the significant reduction in Treg populations observed in *Ifnar1*^−/−^ mice, we further investigated whether Th17 subsets were reciprocally elevated due to this imbalance. Indeed, we observed a significant induction of CD4^+^ IL-17A^+^ T cells in *Ifnar1*^−/−^ mice treated with *B. fragilis* compared with their WT counterparts (Fig. 4 C), underscoring the critical role of type I IFN signaling in maintaining Treg/Th17 balance in response to the commensal bacteria. To further demonstrate the role of commensal bacteria in IFN responses among Tregs, WT SPF mice were treated with an antibiotic cocktail for 2 wk and we observed a significant reduction of pSTAT1 expression within Foxp3^+^ Tregs and decreased IL-10^+^ among Tregs (Fig. S4, E and F). Consistent with this finding, the depletion of commensal bacteria led to decreased IL-10^+^ among CD11c^+^ cells in the colon, further supporting the notion that intestinal commensals are required for DC-directed Treg responses (Fig. S4 G). Together, this data demonstrates a pivotal role for commensal bacteria and type I IFN signaling in orchestrating T cell responses.

We next aimed to elucidate the specific contribution of IFN signaling directly within Foxp3^+^ Tregs in vivo. Using *Ifnar1*^fl/fl^ × Foxp3-Cre (*Ifnar1*^∆Foxp3^), *Ifnar1*^fl/fl^ × CD11c-Cre (*Ifnar1*^∆CD11c^), and *Ifnar1*^fl/fl^ control mice, we orally administered live *B. fragilis* or PBS for 2 wk and examined intestinal Treg responses. While Foxp3^+^ Treg proportions remained consistent between the groups (Fig. 4 D), we did observe a decrease in proportions of Foxp3^+^RORgt^+^ intestinal Tregs in *Ifnar1*^∆Foxp3^ and *Ifnar1*^∆CD11c^ mice compared with floxed controls (Fig. S4 H). As expected, *B. fragilis*–treated WT control mice exhibited a marked induction of IL-10^+^ Tregs compared with PBS-treated groups (Fig. 4 E), in line with previous findings (Round et al., 2011; Shen et al., 2012; Chu et al., 2016). In contrast, the induction of IL-10 by *B. fragilis* was completely abrogated in *Ifnar1*^∆Foxp3^ mice (Fig. 4 E), demonstrating a critical role for IFN signaling in Foxp3 Tregs. Notably, when we performed these experiments using *Ifnar1*^∆CD11c^ mice, the majority of *Ifnar1*^∆CD11c^ mice maintained IL-10 expression in Foxp3^+^ Tregs. However, a subset of antigen presenting cells may still retain IFN signaling in response to commensal bacteria, resulting in Treg responses to *B. fragilis* (Fig. 4 E). Additionally, *Ifnar1*^∆CD11c^ mice displayed significantly higher levels of IL-17A from CD4^+^ Rorgt^+^ cells compared with PBS-treated counterparts as well as WT controls, suggesting a dysregulated cytokine milieu upon type I IFN deficiency in CD11c^+^ cells (Fig. S4 I). Collectively, this data establishes a distinct role for commensal-driven type I IFN signaling in Treg function and highlights the proinflammatory shift that occurs in a type I IFN–deficient intestinal environment.

**Figure S5.**
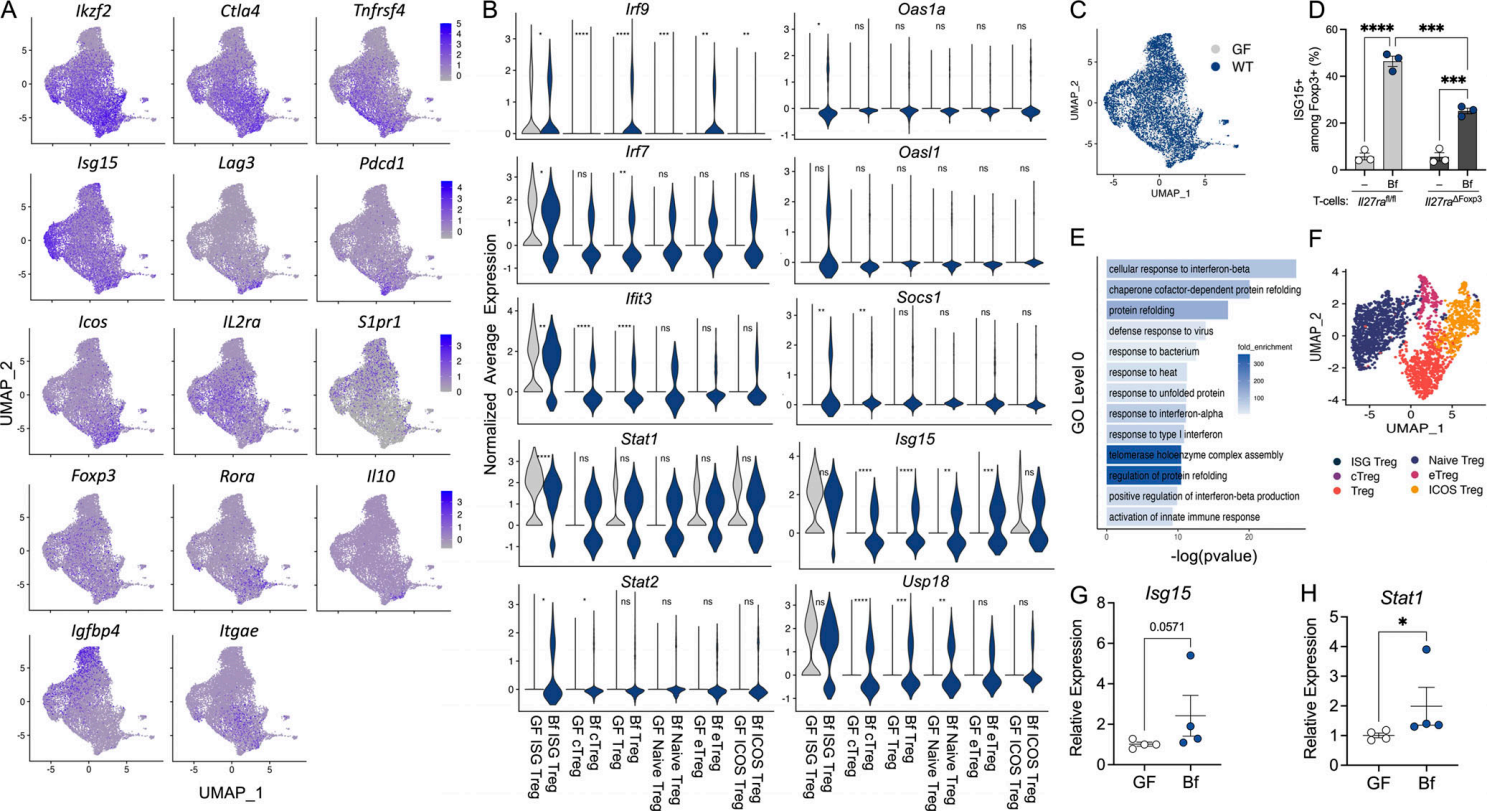
**Colonization with *B. fragilis* promotes intestinal ISG Treg responses.** 4–6-wk-old female GF Foxp3^hCD2^/IL-10^Venus^ mice were orally gavaged with a single dose of *B. fragilis* (Bf; 10^8^ CFU resuspended in sterile PBS). 2 wk later, mice were subjected to 5% DNBS colitis for 3 d and MLNs were harvested, and single suspensions were prepared. CD4^+^hCD2^+^ cells were enriched and subjected to single-cell RNA sequence analysis. **(A)** UMAP projection of genes used as markers to differentiate subpopulations of Tregs. **(B)** Gene expression of *Irf9*, *Oas1a*, *Irf7*, *Oasl1*, *Ifit3*, *Socs1*, *Stat1*, *Isg15*, *Stat2*, and *Usp18* in six clusters of Treg subpopulations: ISG Treg, cTreg, Treg, naive Treg eTreg, and ICOS Treg of GF and Bf mice. **(C)** UMAP projection of single cells colored by GF and Bf populations. **(D)** WT BMDCs were untreated (−) or treated with Bf cocultured with *Il27ra*^fl/fl^ or *Il27ra*^∆Foxp3^ CD4^+^ T cells. Cells were stained and analyzed by flow cytometry for ISG15-producing Foxp3^+^ Tregs. **(E)** KEGG pathway enrichment of ICOS Tregs. **(F)** UMAP of Treg subpopulations from MLNs of GF and Bf mice during steady-state. **(G and H)** MLN CD4^+^ T cells were isolated from GF and Bf-monocolonized mice. Relative expression of (G) *Isg15* and (H) *Stat1* relative to β-actin was evaluated by qRT-PCR. Each point represents a single mouse. Data are representative of at least two independent experiments. Statistical analysis was determined by unpaired *t* test and two-way ANOVA. P < 0.05 (*), P < 0.001 (***), and P < 0.0001 (****).

Recent reports describe a subpopulation of Tregs known as ISG Tregs that expand in mice and humans during inflammatory conditions (Pacella et al., 2020; Sjaastad et al., 2022, *Preprint*). To investigate whether commensal colonization modulates type I IFN–related Treg populations, we next performed single-cell RNA sequencing of GF and *B. fragilis*–monocolonized mice. Notably, we observed a population of Tregs with high expression of ISGs (e.g., *Irf7*, *Irf9*, *Isg15*) from mesenteric lymph nodes (MLNs) during dinitrobenzenesulfonic acid (DNBS)–induced colitis (Fig. 4 F and Fig. S5, A and B). In comparison with GF controls, *B. fragilis*–monocolonized MLNs contained greater numbers of ISG Tregs (14.7% in *B. fragilis* versus 10.8% in GF) and Kyoto Encyclopedia of Genes and Genomes (KEGG) pathway analysis reveals an enrichment of IFN-related pathways, including cellular responses to IFNβ (Fig. 4, F and G; and Fig. S5 C). Interestingly, significant upregulation of genes mediated by IL-27 signaling was observed within the ISG Treg cluster (Fig. 4 G). This is consistent with our in vitro findings, demonstrating the requirement of IL-27 signaling in the induction of *B. fragilis*–mediated ISG15^+^ Tregs (Fig. S5 D). Additionally, we observed higher expression of IFN-related genes such as *Stat1*, *Stat2*, *Irf7*, *Irf9*, *Isg15*, *Ifit3*, and *Oas1a* in ISG Tregs of *B. fragilis*–monocolonized mice (Fig. 4, H–M; and Fig. S5 B). Given that tight regulation of type I IFN is critical for intestinal homeostasis (Katakura et al., 2005; McFarland et al., 2011), we evaluated the expression of various negative regulators of IFN signaling. Of note, *Bst2*, *Socs1*, and *Usp18* showed significant induction in *B. fragilis*–monocolonized mice compared with GF mice during experimental colitis (Fig. S5 B). In addition to ISG Tregs, we also detected a subpopulation of inducible co-stimulator (ICOS) Tregs (Fig. 4, F and M) that also exhibited an enrichment of IFN-related pathways (Fig. S5 E). When we compared the subpopulations of MLN Tregs between steady-state and colitis, we observed that ISG Tregs were less prominent in the absence of intestinal inflammation. Yet, ICOS Tregs were evident in both steady-state and colitis conditions (Fig. 4 F and Fig. S5 F). During steady-state, CD4^+^ T cells isolated from MLNs of GF and *B. fragilis*–monocolonized mice exhibited a modest induction of *Isg15* and *Stat1* in colonized mice, further supporting the specialized role of type I IFN during inflammation (Fig. S5, G and H). Our results here demonstrate that colonization with commensal bacteria promotes the expansion of select subpopulations of Tregs, ISG Treg, and ICOS Tregs during intestinal inflammation. Indeed, a recent study reported a correlation of ISG Tregs to improved outcomes of COVID-19 (Sjaastad et al., 2022, *Preprint*). This suggests a role for the gut microbiota in promoting ISG Treg populations to regulate inflammation—though further work is required to define the precise function of ISG Tregs in the gut.

## Discussion

Efforts to understand how the microbiota primes the IFN system have largely focused on its involvement in antiviral immunity (Steed et al., 2017; Bradley et al., 2019; Schaupp et al., 2020; Stefan et al., 2020; Yang et al., 2021; Erttmann et al., 2022). The role of commensal-induced IFN in promoting intestinal tolerance, however, remains elusive. Several studies have suggested type I IFN and STAT1 (the main transducer of IFN) may be involved in mediating immune tolerance. Type I IFN and STAT1 signaling have been reported to drive expansion of Tregs and promote expression of IL-10 while suppressing intestinal T cells producing IL-17 (Guo et al., 2008; Lee et al., 2012; Stewart et al., 2013; Kole et al., 2013). In contrast, other reports indicate STAT1 and type I IFN induced during viral infections inhibit Treg cell proliferation and activation of Foxp3^+^ Tregs (Ma et al., 2011; Srivastava et al., 2014a, 2014b). These discordant findings highlight the duality of type I IFN responses capable of driving both immunostimulatory and suppressive regulatory functions. Further, these studies indicate the context in which microbes induce IFN may determine whether proinflammatory or tolerogenic immune responses are elicited. We now present findings that support a model whereby commensal bacteria drive autocrine IFN signaling in DCs during steady-state conditions, which is essential for directing Treg development and promoting mucosal homeostasis.

Mounting evidence indicates that intestinal DCs produce and maintain type I IFN in response to the gut microbiota, such that microbiota disruption results in severely diminished type I IFN production (Kole et al., 2013; Kawashima et al., 2013; Stefan et al., 2020). While we primarily focus on CD11c^+^ DCs, we speculate intestinal macrophages and other APCs also mediate this IFN signaling upon sensing commensal bacteria. Recently, several studies demonstrated that commensal bacterium *B. fragilis* induces IFNβ among APCs to trigger systemic antiviral responses (Erttmann et al., 2022; Stefan et al., 2020). Accordingly, we discovered that select human commensal bacteria, including *B. fragilis*, are capable of driving IFNβ production and pSTAT1 signaling in colonic and BMDCs. We further expand these findings by uncovering a critical role of commensal-induced IFN signaling to establish immune tolerance. Mechanistically, we demonstrate that autocrine IFNAR signaling in DCs is important in directing anti-inflammatory responses, as *Ifnar1*^−/−^ DCs and IFNα/β neutralization studies reveal impaired downstream Treg responses. This prompted us to investigate whether tonic IFN induced by commensals poise DCs toward a tolerogenic state. *B. fragilis* treatment of WT DCs led to increased expression of the anti-inflammatory cytokines IL-10 and IL-27. In contrast, *B. fragilis*–treated *Ifnar1*^−/−^ DCs resulted in significantly reduced IL-27, which is known to function downstream of IFNβ signaling (Molle et al., 2007). Of particular interest is the function of IL-27 as an immunoregulatory cytokine that induces IL-10 production to promote the development of tolerogenic Tregs (Yoshida and Hunter, 2015; Kim et al., 2019) and suppresses IL-17 producing T cells to prevent inflammation and autoimmunity (Batten et al., 2006; Kim et al., 2019; Nguyen et al., 2019). We detected significant upregulation in genes related to IL-27–mediated signaling pathways in our single-cell RNA sequencing of gut ISG Tregs from *B. fragilis*–monocolonized mice. We also observed a corresponding increase in the proinflammatory cytokine IL-1β among *Ifnar1*^−/−^ DCs in response to *B. fragilis*. It has been well established that excessive IL-1β leads to the expansion of Th17 cells (Korn et al., 2009). Several studies have demonstrated type I IFN signaling restrains Th17 during autoimmune inflammation and infections (Guo et al., 2008; Prinz et al., 2008; Shinohara et al., 2008). Thus, the reduction in IL-10 and IL-27, along with the proinflammatory shift in IFNAR-deficient DCs, suggests commensal-derived IFN signaling is essential for tolerogenic DC function and mucosal homeostasis.

Our findings indicate that type I IFN signaling is critical for maintaining immune tolerance. Sequencing studies with mice and human CD4^+^ T cells reported an IFN gene signature in Tregs, corroborating our single-cell sequencing data on gut Tregs (Miragaia et al., 2019; Szabo et al., 2019; Sumida et al., 2022). We identified a subpopulation of ISG Tregs expanded in *B. fragilis*–monocolonized mice during intestinal colitis, enriched in expression of IFN-regulation genes (e.g., *Stat1*, *Stat2*, *Irf7*, *Irf9*, *Isg15*, *Usp18*). A recent study reported a population of ISG15 Tregs in human patients where the authors suggest ISG15 is involved in the maintenance of Treg populations during inflammatory conditions such as systemic lupus erythematosus (Pacella et al., 2020). Along these lines, an expansion of ISG Tregs has also been reported during inflammation in the lung, with influenza and COVID-19 infections (Sjaastad et al., 2022, *Preprint*), suggesting the potential role of commensal microbes in limiting inflammation during systemic disease. Furthermore, we observed another Treg subpopulation displaying an IFN gene signature, denoted by its ICOS expression. ICOS^+^ Tregs are conventionally known for their potent induction of IL-10 and maintenance of suppressive function (Löhning et al., 2003; Coquerelle et al., 2009); however, upregulation of this ICOS Treg population upon colonization with *B. fragilis* remained consistent during both steady-state and colitis conditions. Our studies indicate commensal microbes drive IFN signaling within defined subsets of Tregs with potentially distinct roles in maintaining immune homeostasis. Additional work is needed to uncover the function(s) of ISG Tregs and ICOS Tregs during steady-state and inflammation, and whether these two populations exhibit overlapping functions.

The link between the microbiota and type I IFN has been well documented over the last decade. The specific bacterial strains associated with IFN signaling differ among these studies. Yet, it is important to note the effect of the microbiota is not dependent on a single species, but rather, a diverse community of microbes can recapitulate type I IFN responses. In investigating the induction of type I IFN activity among commensal and pathogenic bacteria, we found that pathogenic bacteria yielded significantly higher levels of type I IFN compared with commensal microbes. Moreover, we found a range of induction can be observed among treatments with commensal bacteria and that *B. fragilis* drives the expression of negative regulators of type I IFN signaling. Our study here supports a model whereby commensal bacteria fine-tune type I IFN to orchestrate immune regulation. Of note, STAT1 and IFN signaling have been implicated in promoting both gut homeostasis and driving intestinal inflammation (Cho and Kelsall, 2014; Giles et al., 2017). One potential explanation for this divergent effect may be that both type I (IFNα/β) and type II (IFNɣ) IFN share many of the same signaling components, including STAT1. As such, commensal and pathogenic microbes may signal through a distinct subset of pattern recognition receptors. In turn, this may calibrate different levels of type I and type II IFNs that will ultimately drive either a tolerogenic or inflammatory immune response. While the present study describes a system for the commensal *B. fragilis* to direct immune tolerance in the gut, more broadly our findings can be extended to general mechanisms of immune regulation mediated by the microbiota. For instance, the contribution of the microbiota to responsiveness to immune checkpoint inhibitors (Vétizou et al., 2015; Routy et al., 2018; Matson et al., 2018; Lam et al., 2021) may be linked to the regulation of type I IFN signaling. In line with this notion, *B. fragilis* has been associated with improved response to immune checkpoint blockade in cancer patients (Vétizou et al., 2015)—perhaps owing to its IFN-promoting functions demonstrated here. These observations provide strong evidence that commensal bacteria regulate host immune response through type I IFN pathways.

## Materials and methods

### Mice

SPF WT C57BL/6J (Stock No. 000664), *Ifnar1*^−/−^ (Stock No. 028288), *Tlr2*^*−/−*^ (Stock No. 004650), *Tlr4*^*−/−*^ (Stock No. 029015), and B6.129-*Ifnβ1*^tm1Lky^ (Stock No. 010818) were purchased from The Jackson Laboratory. *Nod2*^fl/fl^ mice were obtained from G. Nunez (University of Michigan, Ann Arbor, MI, USA; Kim et al., 2016) and crossed with CD11c-Cre mice (Stock No. 008068; The Jackson Laboratory) to generate conditional knockout mice. Mice were maintained on Cre^+/−^ × Cre^−/−^ breeding system. *Il27*^fl/fl^ × CD11c-Cre mice were obtained from L-F. Lu (University of California, San Diego, La Jolla, CA, USA). *Il27ra*^fl/fl^ × Foxp3-Cre and × CD11c Cre mice were obtained from B. Min (Northwestern University, Chicago, IL, USA; Do et al., 2017). GF C57BL/6J were bred and housed in flexible film isolators. Foxp3^hCD2^/IL-10^Venus^ were obtained from K. Honda (Keio University, Tokyo Japan; Atarashi et al., 2013) and rederived GF.

For gnotobiotic and monocolonization experiments, 4–6-wk-old male and female GF mice were orally gavaged with a single dose of *B. fragilis* (10^8^ CFU resuspended in sterile PBS). Monocolonized mice were housed in autoclaved cages with autoclaved chow and drinking water supplemented with 100 µg/ml of gentamicin (VetOne). *B. fragilis* strains used are naturally resistant to gentamicin. Fresh fecal samples were collected weekly during cage changing for plating on selective media and GF or *B. fragilis* colonization status was confirmed by PCR. Mice were IP injected with 100 µg/mouse of poly I:C (Sigma-Aldrich) and housed for 4 h.

All procedures were performed in accordance with the guidelines and approved protocols from the Institutional Animal Care and Use Committee of UC San Diego.

### Bacteria

*B. fragilis* (NCTC9343), *B. vulgatus* (ATCC 8482), *Anaerostipes caccae* (DSMZ 14662), *B. thetaiotaomicron* (DSMZ 2079), *Bifidobacterium longum* (NCC 2705), *Blautia producta* (DSMZ 2950), *Clostridium ramosum* (DSMZ 1402), and *L. plantarum* (DSMZ 20174) were grown in Brain Heart Infusion (BHI; Thermo Fisher Scientific) media supplemented with 0.5 μg/ml vitamin K (Sigma-Aldrich) and 0.5% hemin (Sigma-Aldrich) for 72 h in anaerobic conditions at 37°C. Colonies were inoculated into BHI broth supplemented with vitamin K and hemin to grow overnight in anaerobic conditions (10% H_2_, 10% CO_2_, 80% N2; Coy Lab Products) at 37°C. Colonies were inoculated into liquid BHI-S to grow anaerobically for 16–18 h, then subcultured and grown to an OD_600_ of 0.3 (equivalent of 2.1 × 10^8^ CFU/ml) where cells were then heat killed at 90°C for 40 min and used for treatment. *Escherichia coli* K-12 (MG1655), *Salmonella enterica* serovar Typhimurium (IR715), and Group B Streptococcus (NCTC 10/84) were grown in Luria broth media, and *Listeria monocytogenes* (10143S) and *Staphylococcus aureus* (ATCC29213) were grown in BHI aerobically in a shaking incubator at 37°C.

### Antibiotic treatment for the depletion of gut microbiota

For experiments involving depletion of gut microbiota, SPF mice were treated with an antibiotic cocktail for 2–3 wk. Ampicillin (1 g/L; Sigma-Aldrich), metronidazole (0.5 g/L; Thermo Fisher Scientific), neomycin (1 g/L; Thermo Fisher Scientific), and vancomycin (0.5 g/L; VWR) were administered in the drinking water supplemented with 1% Kool-Aid (wt/vol) ad libitum. Control mice were provided 1% Kool-Aid (wt/vol) drinking water without antibiotic cocktail. Fecal samples were collected, plated, and incubated in aerobic and anaerobic conditions for 48–72 h to confirm depletions of gut microbiota.

### DNBS colitis

#### SPF mice

2–3 wk prior to mouse colitis experiments, WT and *Ifnar1*^−/−^ mice were cohoused. WT and *Ifnar1*^−/−^ mice were orally gavaged with PBS or *B. fragilis* (2 × 10^8^ CFU) every other day for 1 wk prior to DNBS (Sigma) administration. On day 7, mice were anesthetized with isoflurane and rectal administration of 5.0–5.5% DNBS in 50% ethanol was applied through a 3.5F catheter (Instech Solomon), as previously described (Chu et al., 2016). Control groups received 50% ethanol (Sham). Briefly, a flexible silicone catheter was inserted 4 cm into the colon and the mice were held in a vertical position for at least 1 min after rectal administration. Mice were monitored and weighed daily for a duration of 72 h. Upon sacrifice, gut tissue was harvested and cLP cells were isolated for further analysis.

#### GF and *B. fragilis*–monocolonized mice

4–5-wk-old female GF mice were orally gavaged with a single dose of *B. fragilis* (10^8^ CFU resuspended in sterile PBS) and colonized for 2 wk prior to DNBS colitis induction. GF and monocolonized mice were housed in autoclaved cages with autoclaved chow and drinking water supplemented with 100 µg/ml of gentamicin (VetOne). Mice were anesthetized with isoflurane and rectal administration of 5.0% DNBS in 50% ethanol was applied through a 3.5F catheter (Instech Solomon), as previously described (Chu et al., 2016). Briefly, a flexible silicone catheter was inserted 4 cm into the colon and the mice were held in a vertical position for at least 1 min after rectal administration. Upon sacrifice, gut tissue was harvested and cLP cells were isolated for single-cell RNA sequencing.

### RNA isolation and quantitative real-time RT-PCR

Cells were harvested, washed, and immediately lysed in RNA lysis (RLT) buffer for RNA isolation using the RNeasy Mini Kit according to the manufacturer’s protocol (Qiagen). Tissue was harvested and placed in RNA*later* (Invitrogen), homogenized, and lysed according to the manufacturer’s protocol (Qiagen). 1 µg of RNA was reversed transcribed using SuperScript IV Reverse Transcriptase according to the manufacturer’s protocol (Thermo Fisher Scientific) and diluted to 10 µg/µl based on the input concentration of total RNA. Gene-specific primers were synthesized by Integrated DNA Technologies and sequences are provided in Table 1. Real-time PCR was performed on cDNA using the QuantStudio (Thermo Fisher Scientific).

**Table 1.** Primers used for real-time PCR

Gene	Forward (5′ ➨ 3′)	Reverse (5′ ➨ 3′)	Reference
*Mx1*	TGT​GGA​CAT​TGC​TAC​CAC​AGA	AAG​GCA​GTT​TGG​ACC​ATC​TC	Uccellini and García-Sastre (2018)
*Mx2*	TGG​CAC​TTC​CAG​TTC​CTC​TCA	GGT​TGT​GAG​CCT​CTT​GGC​GG	This study
Pan-*Ifna*	CCT​GAG​GAA/GAG​AAG​AAA​CAC​AGC​C	GGC​TCT​CCA​GAC/TTT​CTG​CTC​TG	Tailor et al. (2007)
*Ifnb*	CAG​CTC​CAA​GAA​AGG​ACG​AAC	GGC​AGT​GTA​ACT​CTT​CTG​CAT	Primerbank ID: 6754304a1
*Ifng*	CCT​TCT​TCA​GCA​ACA​GCA​AGG​C	GAC​TCC​TTT​TCC​GCT​TCC​TGA​G	Winer et al. (2001)
*Oas1*	ATG​GAG​CAC​GGA​CTC​AGG​A	TCA​CAC​ACG​ACA​TTG​ACG​GC	Uccellini and García-Sastre (2018)
*Irf3*	CGG​AAA​GAA​GTG​TTG​CGG​TTA​GC	CAG​GCT​GCT​TTT​GCC​ATT​GGT​G	Pang et al. (2018)
*Irf7*	GCC​AGG​AGC​AAG​ACC​GTG​TT	TGC​CCC​ACC​ACT​GCC​TGT​A	Fensterl et al. (2008)
*Irf9*	TAA​CCG​CTT​GCC​CTG​CAA​CT	ACT​TTG​CCT​GAG​GCC​AAT​CCT​GA	This study
*Isg15*	CTG​ACT​GTG​AGA​GCA​AGC​AGC	ACC​TTT​AGG​TCC​CAG​GCC​ATT​G	This study
*Ifit1*	CAG​AAG​CAC​ACA​TTG​AAG​AA	TGT​AAG​TAG​CCA​GAG​GAA​GG	Fensterl et al. (2008)
*Stat1*	TCA​CAG​TGG​TTC​GAG​CTT​CAG	GCA​AAC​GAG​ACA​TCA​TAG​GCA	Uccellini and García-Sastre (2018)
*Il1b*	CAG​GCA​GGC​AGT​ATC​ACT​CAT​TG	CCA​GCA​GGT​TAT​CAT​CAT​CAT​CCC	This study
*Il10*	GGT​TGC​CAA​GCC​TTA​TCG​GA	ACC​TGC​TCC​ACT​GCC​TTG​C	Wagage et al. (2014)
*Il6*	AAA​GAC​AAA​GCC​AGA​GTC​CTT​CAG	TGG​TCC​TTA​GCC​ACT​CCT​TCT​GTG	This study
*Il12p35*	TAC​TAG​AGA​GAC​TTC​TTC​CAC​AAC​AA	TCT​GGT​ACA​TCT​TCA​AGT​CCT​CAT​AG	This study
*Il12p40*	CTC​AGA​AGC​TAA​CCA​TCT​CCT​GG	CAC​AGG​TGA​GGT​TCA​CTG​TTT​C	Liu et al. (2013)
*Il27p28*	GGC​CAT​GAG​GCT​GGA​TCT​C	AAC​ATT​TGA​ATC​CTG​CAG​CCA	Maroof and Kaye (2008)
*Il17a*	CTC​CAG​AAG​GCC​CTC​AGA​CTA	GGGTCTTCATTGCGGTGG	Hod-Dvorai et al. (2011)
*Tnfa*	ACG​GCA​TGG​ATC​TCA​AAG​AC	GTG​GGT​GAG​GAG​CAC​GTA​GT	Okazaki et al. (2016)
*Actb*	GCT​GAG​AGG​GAA​ATC​GTG​CGT​G	CCA​GGG​AGG​AAG​AGG​ATG​CGG	Bronner et al. (2018)

### Colon explant cultures

1 cm of colon was cut from tissue cleaned with 1× PBS. The tissue was placed in 500 µl of complete RPMI 1640 (10% fetal bovine serum, 50 U/ml penicillin, 50 µg/ml streptomycin, 2 mm L-glutamine, 1 mm sodium pyruvate, 1 mm HEPES, non-essential amino acids, and β-mercaptoethanol; Gibco) and cultured for 18–24 h with or without stimulation with poly I:C (Sigma-Aldrich) at 37°C and 5% CO_2_. Supernatant was then collected and stored at −20°C and used for quantification of cytokine secretion by ELISA.

### In vitro DC:T cell coculture

Coculture of BMDCs and CD4^+^ T cells were performed previously described (Chu et al., 2016). Briefly, BMDCs were generated from bone marrow progenitor cells isolated from femurs of WT or IFNAR1-deficient mice in the presence of 20 ng/ml GM-CSF (Miltenyi) in complete RPMI 1640 (10% fetal bovine serum, 50 U/ml penicillin, 50 µg/ml streptomycin, 2 mm L-glutamine, 1 mm sodium pyruvate, 1 mm HEPES, non-essential amino acids, and β-mercaptoethanol). BMDCs were pulsed with PBS, *B. fragilis* at multiplicity of infection (MOI) of 1 or 5 for 18–24 h. BMDCs were washed and cocultured with splenic CD4^+^ T cells at a ratio of 1:10 (DC:CD4+ T cells) in the presence of anti-mouse CD3 (eBiosciences), mouse IL-2 (Peprotech), and human TGF-β (Peprotech). Cocultures were treated with 10 µg/ml of anti-Mouse IFNα and anti-Mouse IFNβ (Leinco Technologies) every 24 h. After 3 d of coculture, supernatants were collected and stored at −20°C for ELISAs, and cells were stained with specific antibodies and viability dye for analysis by flow cytometry.

### Flow cytometry and ELISA

Cells were stained for 30 min at 4°C with either LIVE/DEAD fixable violet or yellow dead stain kit (Life Technologies), with empirically titrated concentrations of the following antibodies: PE-Cy7-conjugated anti-mouse CD4 (clone: RM4-5), PE-conjugated anti-human CD2 (clone: RPA-2.10), APC-conjugated anti-mouse CD25 (clone: PC61.5), BV785-conjugated CD11c (clone: N418), PE-Cy7-conjugated anti-mouse Siglec H (clone: eBio440c), and eF660-conjugated anti-GFP (clone: 5F12.4). For intracellular staining, cells were fixed and permeabilized using the Transcription Factor Phospho Buffer Set (BD Pharmigen) according to the manufacturer's protocol. Intracellular staining was performed with the following antibodies: AlexaFluor647-conjugated anti-mouse pSTAT1 (clone: A15158B), PerCP-eF710-conjugated anti-mouse RORγ (t; clone: AFKJS-9), PE-Dazzle594-conjugated anti-mouse Tbet anti-mouse (clone: 4B10), PE-conjugated anti-mouse IL-10 (clone: JES5-16E3), PE-Cy7-conjugated anti-mouse IL-17 (clone: eBio17B7), BV785-conjugated anti-mouse IFNg (clone: XMG1.2), unconjugated anti-mouse ISG15 (polyclonal), APC Goat anti-mouse IgG (clone: Poly4053), and/or FITC-conjugated anti-mouse Foxp3 (clone: FJK-16s) for 3–4 h. All antibodies were purchased from Thermo Fisher Scientific/eBiosciences, BD, and Biolegend. Cell acquisition was performed on FACSCelesta (BD), and data were analyzed using FlowJo software suite (TreeStar). For ELISAs, cell supernatants from DC or DC:T cell cocultures were collected and measured using a commercially available kit for IL-10 (Thermo Fisher Scientific/eBiosciences), IFNβ (PBL Assay Science), and antiviral response analytes (Biolegend).

### Isolation of cells from tissues

MLNs were processed by mashing tissues through a 100-µm cell strainer (BD Falcon) to generate single-cell suspensions. Colon tissues were cut open longitudinally and luminal contents were flushed with cold PBS. Colon tissues were cut into 1-cm pieces and incubated in 10 mm dithiothreitol with gentle shaking at 37°C for 20 min, followed by incubation in 20 mm EDTA for 20 min. Supernatant was removed and the remaining tissues were incubated in 1 mg/ml Collagenase D and 0.5 mg/ml DNase I. cLP cells were filtered through a 70-µm cell strainer and separated by 40%:80% Percoll density gradient. Enrichment of CD4^+^, CD11c^+^, and Foxp3-hCD2^+^ cells was performed using magnetic activated cell sorting beads (Miltenyi).

### Ex vivo cultures

Lymphocytes isolated from the spleen or cLP were cultured in complete RPMI 1640 (10% fetal bovine serum, 50 U/ml penicillin, 50 µg/ml streptomycin, 2 mm L-glutamine, 1 mm sodium pyruvate, 1 mm HEPES, non-essential amino acids, and β-mercaptoethanol). Splenocytes were treated with *B. fragilis* at MOI of 10. Colon cells were either untreated or treated with 25 µg/ml recombinant mouse IFN-β1 (Biolegend). Supernatant was collected for ELISA and cells were stained for flow.

### Single-cell RNA sequencing

MLNs were generated and ∼8,000–10,000 cells were loaded onto a Chromium Single Cell Chip (10× Genomics), according to the manufacturer’s protocol. mRNA was barcoded during cDNA synthesis and pooled for Illumina sequencing. Libraries were sequenced with an eight-base index read on a Novaseq 6000. FASTQ files were demultiplexed and aligned to the mouse reference genome (v3.0.2; Cell Ranger 10× Genomics). Analysis was performed using Loupe (10× Genomics). The resulting feature-barcode matrices were analyzed with the Seurat R package v3.1.5 (Hao et al., 2021). Cells were filtered to remove cells that expressed <200 genes and cells with >5% expression of mitochondrial genes. The counts were normalized by total read count and log-transformed. Features were counted with the “vst” method to identify 2,000 genes from each sample that had high variation. Each of the samples was scaled and clustered at a 0.5 resolution, and the clustering was visualized with Uniform Manifold Approximation and Projection (UMAP). CellKB was used to identify each cluster (Patil and Patil, 2022, *Preprint*). The Treg population was subsetted and reclustered. The clusters were identified by marker genes (Fig. S5 A). The average expression of each gene was calculated per the sample type (GF or *B. fragilis*) and per cluster. Gene Ontology (GO) analysis was performed for the genes upregulated in the *B. fragilis* ISG Treg population compared with the GF ISG Treg population by conducting a differential expression analysis between these two populations and selecting the genes with a log fold change of >0.25. This list of genes was annotated using the GO web server using Panther (Thomas et al., 2022). The resulting xml file was imported and analyzed with R (R Core Team, 2013). Feature plots and violin plots were made using Seurat. The heatmap was made by selecting the top 100 differentially expressed genes between the GF and *B. fragilis* groups. Ribosomal genes were filtered out.

### Statistical analysis

Student’s *t* test was used for pairwise comparisons. One-way and two-way ANOVA with Post-hoc Tukey test were used for comparisons among one or more groups, respectively, using the GraphPad PRISM software. P value <0.05 was considered significant.

### Online supplemental material

Fig. S1 shows the expression of *Ifnar1* among GF, *B. fragilis*–monocolonized, and SPF mice; proportion of CD11c^+^ cells and pSTAT1 expression among CD11c^+^ cells from antibiotics treated or untreated mice; heatmap of cytokines and chemokines among GF and SPF mice with or without poly I:C treatment; and expression of type I IFN–related genes by quantitative RT-PCR (qRT-PCR) from GF, *B. fragilis*–monocolonized, and SPF mice with or without poly I:C treatment. Fig. S2 shows pSTAT1 expression among BMDCs treated with commensal or pathogenic bacteria; IFN-β secretion measured from the supernatant of BMDCs treated with pathogens; expression of type I IFN–related genes by qRT-PCR among WT or *Ifnar1*^*−/−*^ BMDCs with or without *B. fragilis* treatment. IFN-β expression from WT or NOD2, TLR2, or TLR4 knockout BMDCs with or without *B. fragilis* treatment; and secretion of IL-10 and IFNβ and expression of pSTAT1 evaluated among *Il27*^*fl/fl*^ or *Il27*^∆CD11c^ BMDCs with or without *B. fragilis* treatment. Fig. S3 shows expression and secretion of both cytokines and chemokines by qRT-PCR and ELISA, respectively, among WT and *Ifnar1*^*−/−*^ BMDCs treated with *B. fragilis*. Fig. S4 shows induction of IL-10^+^ Tregs upon treatment of CD4^+^ T cell with IL-27 in vitro; expression of IL-10^+^ among Tregs and secretion of IL-27 among BMDCs upon treatment with commensal bacteria; IL-27 production among CD4^+^ *Il27ra*^fl/fl^ or *Il27ra*^∆Foxp3^ T-cells co-cultured with WT or *Ifnar1*^*−/−*^ BMDCs treated with *B. fragilis*; expression of Foxp3^+^ and pSTAT1 among Foxp3^+^ Tregs upon antibiotic treatment of SPF mice; expression of IL-10^+^ among Foxp3^+^ Tregs and CD11c^+^ cells upon antibiotic treatment of SPF mice; population percentages of Foxp3^+^RORgt^+^ and IL-17A^+^ among RORgt^+^ T cells among *Ifnar1*^*fl/f*^,* Ifnar1*^∆CD11c^, and *Ifnar1*^∆Foxp3^ mice gavaged with either PBS or *B. fragilis*; and colon length and change in weight percentages among DNBS induced and EtOH control GF of *B. fragilis*–monocolonized mice. Fig. S5 shows UMAP projection of genes used as markers to differentiate subpopulations of Tregs; gene expression of *Irf9*, *Oas1a*, *Irf7*, *Oasl1*, *Ifit3*, *Socs1*, *Stat1*, *Isg15*, *Stat2*, and *Usp18* in six clusters of Treg subpopulations; UMAP projection of single cells colored by GF and *B. fragilis* populations; ISG15 production among *Il27ra*^fl/fl^ or *Il27ra*^∆Foxp3^ CD4^+^ T cells cocultured with WT BMDCs untreated or treated with *B. fragilis*; KEGG pathway enrichment of ICOS Tregs; UMAP of Treg subpopulations from MLNs of GF and *B. fragilis* mice during steady-state; and relative expression of *Isg15* and *Stat1* from CD4^+^ T cells from MLNs of GF and *B. fragilis*–monocolonized mice.

## Data Availability

The data are available from the corresponding author upon reasonable request. The single-cell RNA sequencing data underlying Fig. 4 are openly available in the NCBI Gene Expression Omnibus (GEO) under accession number GSE248021.
